# Tumor suppressor network dysregulation in neuroblastoma: molecular mechanisms and precision therapeutic opportunities

**DOI:** 10.3389/fcell.2026.1844582

**Published:** 2026-06-19

**Authors:** Rohan Gupta, Sorabh Lakhanpal, Naveen Kumar, Rupak Nagraik, Karthikeyan Ravi, Shivanshu Sharma, Mosleh Mohammad Abomughaid, Smita Kumari, Niraj Kumar Jha

**Affiliations:** 1 Department of Biotechnology and Bioengineering, School of Biosciences and Technology, Galgotias University, Greater Noida, Uttar Pradesh, India; 2 School of Pharmaceutical Sciences, Lovely Professional University, Phagwara, Punjab, India; 3 Department of Physics, School of Basic and Applied Sciences, Galgotias University, Greater Noida, Uttar Pradesh, India; 4 Department of Biotechnology, Graphic Era Deemed to Be University, Dehradun, Uttarakhand, India; 5 Centre for Herbal Pharmacology and Environmental Sustainability, Chettinad Hospital and Research Institute, Chettinad Academy of Research and Education, Kelambakkam, Tamil Nadu, India; 6 School of Pharmacy, Sharda University, Greater Noida, Uttar Pradesh, India; 7 Department of Medical Laboratory Sciences, College of Applied Medical Sciences, University of Bisha, Bisha, Saudi Arabia; 8 College of Pharmacy, The Ohio State University, Columbus, OH, United States

**Keywords:** epigenetic dysregulation, neuroblastoma, precision oncology, targeted therapy, tumor suppressor genes

## Abstract

Neuroblastoma is the most common extracranial solid malignancy in children and accounts for nearly 15% of paediatric cancer-related mortality, underscoring its substantial clinical burden. Although multimodal therapeutic strategies, including chemotherapy, surgical resection, radiotherapy, stem cell transplantation, and immunotherapy, have improved outcomes in low- and intermediate-risk disease, survival rates for high-risk neuroblastoma remain poor due to frequent relapse and treatment resistance. While oncogenic drivers, such as *MYCN* amplification and *ALK* mutations have been extensively investigated, accumulating genomic and epigenomic evidence indicates that disruption of tumor suppressor gene (TSG) networks plays a central role in neuroblastoma pathogenesis. Unlike many adult malignancies driven by somatic mutations, neuroblastoma frequently exhibits tumor suppressor gene dysfunction through chromosomal deletions, copy number alterations, epigenetic silencing, and dysregulated signaling pathways. Major tumor suppressive pathways affected include Tp53-mediated apoptosis and genomic stability, RB-dependent cell cycle regulation, PTEN/PI3K/AKT survival signaling, Hippo pathway control of proliferation and stemness, and DNA damage response mechanisms. These interconnected networks drive tumor progression, metastatic dissemination, immune evasion, metabolic adaptation, and therapeutic resistance. Consequently, researchers are actively exploring therapeutic strategies targeting tumor suppressor-associated vulnerabilities. However, clinical translation remains challenging due to tumor heterogeneity, developmental toxicity concerns, and adaptive resistance mechanisms. This review summarizes the molecular mechanisms underlying tumor suppressor dysfunction in neuroblastoma and discusses emerging translational strategies targeting interconnected oncogenic, epigenetic, metabolic, and immune-associated signaling networks.

## Graphical Abstract

**Figure:**
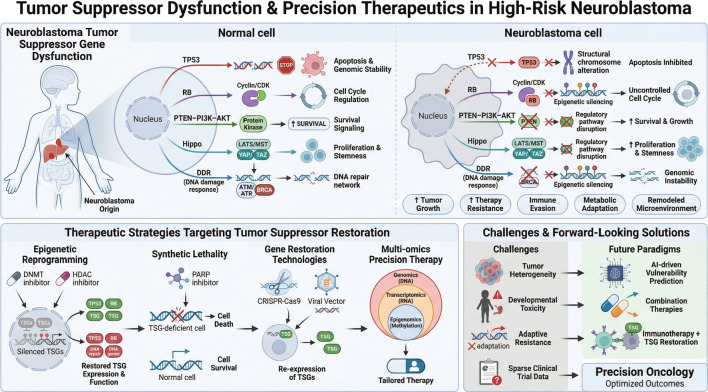
Neuroblastoma is the most frequent solid tumor outside the central nervous system in children and is a leading cause of cancer-related mortality in children, especially among high-risk groups. Unlike most adult cancers, neuroblastoma is primarily caused by the dysfunction of tumor suppressor genes due to chromosomal instability, epigenetics, and signaling pathway disruptions. The key pathways involved include TP53, RB, PTEN/PI3K/AKT, Hippo, and those regulating the DNA damage response. Current and future directions in therapy target epigenetic reactivation, synthetic lethality (PARP inhibitors), functional restoration, and precision medicine based on multi-omics analysis. However, several challenges remain, including intratumoral heterogeneity, toxicity, resistance, and the current paucity of clinical trials in pediatric populations. Future directions include the use of artificial intelligence to guide therapeutic decisions and combination therapies to improve outcomes.

## Highlights

- Neuroblastoma TSG loss driven by structural and epigenetic alterations.
- TP53, RB, PTEN, Hippo, DDR pathways frequently inactivated.
- TSG dysfunction promotes proliferation, resistance, and metastasis.
- Epigenetic reactivation and synthetic lethality offer therapeutic promise.
- Multi-omics enables precision targeting of tumor suppressor vulnerabilities.

## 1 Introduction

Neuroblastoma is a childhood malignancy originating from neural crest-derived progenitor cells of the sympathetic nervous system and represents one of the most clinically heterogeneous paediatric solid tumors. Disease behavior ranges from spontaneous regression in infants to highly aggressive metastatic disease associated with poor survival in high-risk patients (Katta et al., 2023; Sainero-Alcolado et al., 2024). Although multimodal treatment strategies including chemotherapy, surgery, radiotherapy, immunotherapy, and differentiation therapy have improved outcomes in selected patient groups, relapse and treatment resistance remain major causes of therapeutic failure in high-risk neuroblastoma (Mabe et al., 2022; Grossmann et al., 2024; Yamamoto et al., 2025; Yu et al., 2025). Recent molecular and genomic analyses improve understanding of neuroblastoma biology. Unlike many adult cancers, neuroblastoma tumors are characterized by a relatively low number of recurrent point mutations. However, tumor pathogenesis is primarily fuelled by structural genomic variations, segmental chromosomal changes, copy number variations, and massive epigenetic remodeling (van Groningen et al., 2017; Fransson et al., 2020; Barford et al., 2023; Epp et al., 2023; Abu-Zaid et al., 2024). These changes often impair TSG pathways involved in cell cycle regulation, apoptosis, differentiation, and genomic integrity. Further, multiple mechanisms drive tumor suppressor inactivation, including chromosomal deletions, promoter hypermethylation, regulation by non-coding RNAs (ncRNAs), and post-transcriptional modifications (PTMs). These molecular alterations collectively promote uncontrolled tumor growth (Lázcoz et al., 2006; Fujita et al., 2008; Bagci et al., 2023; Liu et al., 2024; Thombare et al., 2024).

Importantly, recent advances in genomic, epigenetic, and transcriptomic profiling have improved understanding of how *MYCN* amplification cooperates with tumor suppressor dysfunction to drive neuroblastoma progression, therapeutic resistance, and tumor microenvironment remodeling. *MYCN* overexpression promotes transcriptional and epigenetic reprogramming associated with chromosomal instability, replication stress, metabolic adaptation, and suppression of differentiation-associated pathways (King et al., 2020). Experimental evidence further demonstrates that *MYCN* amplification cooperates functionally with dysregulation of Tp53 pathway regulators, *PTEN*, *CASZ1*, *RASSF* family members, and chromosome 1p/11q-associated tumor suppressors to accelerate aggressive tumor behavior and therapeutic resistance. In addition, *MYCN*-driven recruitment of chromatin remodeling complexes, histone-modifying enzymes, and DNA methyltransferases contributes to epigenetic repression of multiple tumor suppressor loci and disruption of DDR pathways, collectively promoting highly aggressive neuroblastoma phenotypes (Shohet, 2012; Gherardi et al., 2013; Hu et al., 2019). Moreover, advances in high-throughput sequencing and multi-omics technologies have facilitated identification of tumor-specific molecular vulnerabilities and interconnected oncogenic signaling networks in neuroblastoma. Emerging translational strategies targeting epigenetic dysregulation, synthetic lethal dependencies, DDR pathways, and tumor suppressor-associated signaling networks are currently being actively investigated. However, intratumoral heterogeneity, clonal evolution, adaptive signaling rewiring, and tumor microenvironment interactions continue to represent major barriers to durable therapeutic responses and successful clinical translation (Roeschert et al., 2021; Gundem et al., 2023; Borenäs et al., 2024). In this context, understanding the molecular mechanisms underlying tumor suppressor dysregulation may provide important insights into neuroblastoma progression and reveal novel therapeutic vulnerabilities. This review focuses on the molecular mechanisms of TSG alteration in neuroblastoma, associated translational challenges, and emerging therapeutic opportunities targeting interconnected oncogenic, epigenetic, metabolic, and immune-associated signaling pathways.

## 2 TSG landscape in neuroblastoma: structural and genomic context of TSG loss

**FIGURE 1:**
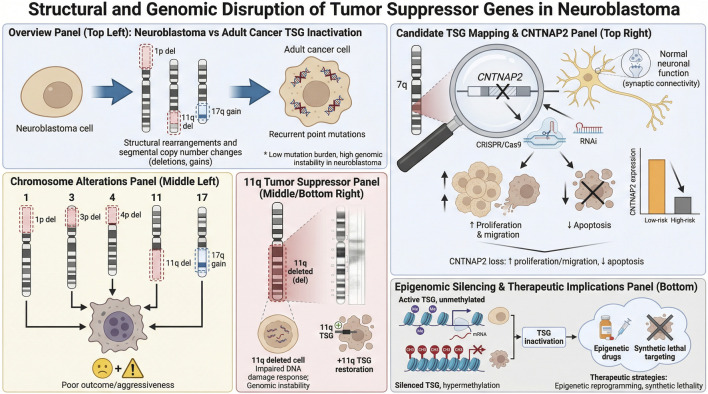
Structural and genomic basis for the disruption of TSGs in neuroblastoma: Neuroblastoma is marked by a small number of point mutations coupled with a high degree of genomic instability, including frequent deletions at 1p, 3p, 4p, and 11q, and 17q gain. The mapping of candidate TSGs has identified the functional inactivation of *CNTNAP2*, which supports cell proliferation and migration while reducing apoptosis. The above schematic also illustrates the disruption of the DDR pathway due to the loss of 11q TSGs and the epigenetic inactivation of TSGs by promoter hypermethylation.

The genomic profile of TSG alterations in neuroblastoma is unique among many adult cancers. Unlike many adult malignancies, neuroblastoma is not fuelled by point mutations but rather by large-scale chromosomal rearrangements, segmental copy number changes, and genomic structural variations that globally reshape the tumor suppressor genome. Large-scale genomic sequencing studies, such as whole genome sequencing (WGS) and single nucleotide polymorphism (SNP) array analysis consistently demonstrate that high-risk neuroblastoma tumors have a low somatic mutation burden but are globally unstable (Janoueix-Lerosey et al., 2009; Molenaar et al., 2012; Pugh et al., 2013; Peifer et al., 2015; Lopez et al., 2020; Chen et al., 2025). Common structural changes include deletions of chromosome arms 1p, 3p, 4p, and 11q, as well as gains of 17q, which are strongly associated with poor disease phenotypes and outcomes. Further, genomic mapping studies have identified candidate TSGs in these regions. For instance, high-resolution WGS analysis of primary neuroblastoma tumors has revealed recurrent structural alterations in genes regulating neuronal function and adhesion molecules, such as Contactin-associated protein-like 2 (*CNTNAP2*), which is involved in neuronal cell-to-cell communication and synaptic structure (Molenaar et al., 2012; Geng et al., 2024; Liu et al., 2026). Additionally, functional studies using CRISPR-mediated gene knockdown and RNA interference (RNAi) in neuroblastoma cell lines have shown that the loss of *CNTNAP2* expression is associated with enhanced proliferation, migration, and apoptosis resistance, thus confirming the tumor suppressor function of *CNTNAP2*. Moreover, the gene expression analysis of patient tumor samples shows that there is a significantly lower expression of *CNTNAP2* in high-risk neuroblastoma patients compared to low-risk patients or those with regressing tumors, indicating that structural disruption rather than point mutation is responsible for the loss of *CNTNAP2* function (Liu et al., 2026). Evidence also supports the role of chromosome 11q deletions in the disruption of tumor suppressor pathways. For instance, cytogenetic studies and array comparative genomic hybridization (aCGH) analysis suggest that 11q deletion is associated with aberrant DDR signaling and increased genomic instability (Plantaz et al., 2001; Mandriota et al., 2015). Functional validation studies also show that the reintroduction of candidate TSGs in the 11q region in neuroblastoma cell lines can reduce tumor growth and increase apoptosis, thus confirming the biological relevance of structural TSG disruption (Keane et al., 2022). Additionally, epigenomic studies using methylation array and chromatin accessibility analysis also suggest that the silencing of tumor suppressors in neuroblastoma is more due to chromatin remodeling and promoter hypermethylation than gene mutation, thus emphasizing the importance of genome architecture in the pathogenesis of the disease (Michalowski et al., 2008; Kiss et al., 2012; Olsson et al., 2016). Recent evidence has additionally identified *SRCIN1* and *CASZ1* as functionally important tumor suppressors in neuroblastoma. Functional studies demonstrate that *SRCIN1* suppresses neuroblastoma cell proliferation, migration, invasion, and epithelial-to-mesenchymal transition through inhibition of SRC-mediated oncogenic signaling pathways (Liu et al., 2011). Similarly, *CASZ1*, a neural crest developmental transcription factor frequently downregulated in high-risk neuroblastoma, promotes neuronal differentiation and inhibits tumor progression. Experimental studies further suggest that coordinated upregulation of *SRCIN1* and *CASZ1* exerts synergistic antitumor activity, leading to enhanced suppression of tumor growth, migration, and metastatic potential. These findings further support the concept that disruption of interconnected tumor suppressor networks, rather than isolated single-gene alterations, contributes significantly to neuroblastoma pathogenesis and therapeutic resistance (Liu et al., 2022). Moreover, beyond structural deletions and epigenetic silencing, emerging evidence indicates that non-coding regulatory variants contribute significantly to tumor suppressor dysregulation in neuroblastoma. Single nucleotide variants (SNVs) located within enhancer elements, promoter regions, transcription factor binding sites, and super-enhancers can disrupt normal transcriptional regulation of TSGs without directly altering coding sequences (Gomez et al., 2022b). Current evidence supports that non-coding variants may contribute to tumor suppressor dysregulation in neuroblastoma through chromatin accessibility, impair enhancer-promoter interactions, and reduce recruitment of lineage-specific transcription factors. Notably, recent studies have identified pathogenic non-coding variants associated with reduced expression of genes, such as *CTTNBP2* and *MCF2L*, supporting the concept that regulatory genomic alterations may function as oncogenic drivers in neuroblastoma (Montella et al., 2025). These findings are particularly important given the relatively low coding mutational burden observed in high-risk neuroblastoma and further emphasize the importance of integrating epigenomic, chromatin conformation, and non-coding genome analyses into precision oncology approaches. Taken together, the data provide evidence for a mechanism of TSG inactivation in neuroblastoma that is mainly fuelled by genome structural disruption and not by conventional mutational inactivation. Structural variations can lead to the translocation of regulatory sequences, disruption of enhancer-promoter contacts, or changes in three-dimensional chromatin conformation, ultimately causing the transcriptional silencing of TSGs. This genome architecture-mediated mechanism of TSG loss has profound therapeutic implications (Wang and Yue, 2024; Dong et al., 2026). It suggests that therapies aimed at correcting chromatin structure, overcoming epigenetic silencing, or targeting synthetic lethality associated with structural deletions could be highly effective in neuroblastoma. Therefore, understanding the structural genomic basis of TSG loss is a crucial step toward the development of precision therapies targeting TSG pathways in neuroblastoma (Figure 1).

## 3 Core tumor suppressor pathways in neuroblastoma

### 3.1 p53 signaling axis

The p53 signaling pathway is a key node in the maintenance of genomic stability, mediating apoptosis, cell cycle arrest, senescence, and DNA repair. In neuroblastoma, *Tp53* mutations are relatively rare at the time of diagnosis, especially in primary tumors, compared to most adult cancers. However, functional inactivation of the *p53* pathway is not rare and occurs through disruption of upstream regulatory circuits rather than direct mutation of the *Tp53* gene. One important mechanism of *p53* pathway inactivation with clinical relevance is the overexpression or amplification of Mouse double minute 2 homolog (*MDM2*), an E3 ubiquitin ligase that enhances proteasomal degradation of *p53* (Carr-Wilkinson et al., 2010; Benchia et al., 2025). Overexpression of *MDM2* has been found in selected patients with high-risk neuroblastoma and is predictive of poor outcome, resistance to therapy, and aggressive disease phenotype. Further, there is clinical and biological evidence that inactivation of the *p53* pathway is a mechanism of chemotherapy resistance. Using neuroblastoma patient-derived xenograft (PDX) models and cell line models, *MDM2* overexpression reduces p53-mediated apoptotic responses to DNA-damaging chemotherapy (Barbieri et al., 2006; Zafar et al., 2021). In addition, relapsed neuroblastoma tumors frequently exhibit increased TP53 pathway inactivation than primary tumors, indicating that selective pressure from therapy leads to functional inactivation of the *p53* pathway. Likewise, high *MDM2* expression is predictive of decreased event-free survival and resistance to conventional chemotherapy, including platinum and topoisomerase inhibitors (Rodriguez-Lopez et al., 2001; Gamble et al., 2012). Moreover, *MYCN* amplification has also been shown to functionally suppress *p53*-mediated apoptotic signaling through enhanced MDM2 activity and adaptation to oncogene-induced replication stress. *MYCN*-amplified neuroblastoma cells increasingly depend on *Tp53* pathway suppression to sustain survival under conditions of persistent proliferative and genotoxic stress, thereby creating selective pressure for functional inactivation of *p53*-mediated checkpoint and apoptotic responses (Gamble et al., 2012).

Recent advances in systems biology and modeling have expanded the scope of *p53* action beyond its intrinsically tumor cell-mediated roles. Multi-omics integration analyses suggest that *p53* signaling modulates tumor microenvironment (TME) interactions, such as immune modulation, metabolic shift, and stromal signaling. These findings are supported by single-cell transcriptomic studies, which have shown diverse *p53* pathway activation in tumor and immune cells and may predict treatment response and recurrence (An et al., 2025; Li et al., 2025; Wang Y. et al., 2025; Xie et al., 2025). In terms of therapeutic applications, the reactivation of *p53* pathway function is a major paradigm in high-risk neuroblastoma. *MDM2* inhibitors, including multi-class compounds and next-generation compounds, such as idasanutlin, have shown the ability to stabilize *p53* and trigger apoptosis in preclinical neuroblastoma models. Similarly, early-phase clinical trials of *MDM2* inhibitors in paediatric solid malignancies have shown biological activity and acceptable safety profiles (Chen et al., 2015; Lakoma et al., 2015; Rubio-San-Simón et al., 2025). Moreover, *p53* reactivation molecules, including small molecules that reactivate mutant or functionally inactivated *p53*, are being investigated in early-phase translational studies. Further, *MYCN* amplification has also been shown to functionally suppress *p53*-mediated apoptotic signaling through enhanced *MDM2* activity and adaptation to oncogene-induced replication stress. Experimental studies suggest that *MYCN*-overexpressing neuroblastoma cells develop increased dependence on *Tp53* pathway suppression to maintain survival under conditions of sustained proliferative and genotoxic stress, thereby contributing to tumor progression and therapeutic resistance.

**FIGURE 2:**
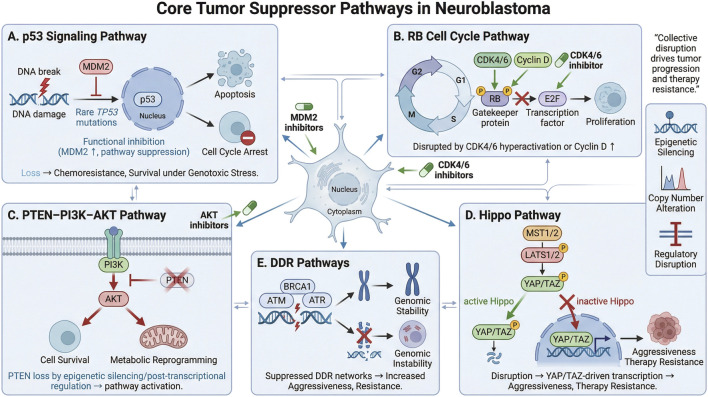
Tumor suppressor pathways involved in neuroblastoma development and resistance to therapies: The pathways are represented by the suppression of the *p53* pathway by *MDM2*, the disruption of the retinoblastoma (RB) pathway by the overexpression of *CDK4/6*, the loss of *PTEN* leading to PI3K/AKT survival signaling, the inactivation of the Hippo pathway and the subsequent transcription by YAP/TAZ, and the inactivation of the DDR pathway. The main approaches being developed for the treatment of neuroblastoma include the use of *MDM2* inhibitors, *CDK4/6* inhibitors, *AKT* inhibitors, and DDR pathway inhibitors.

Combination regimens combining *MDM2* inhibitors with chemotherapy or targeted therapies have shown synergistic antitumor effects in preclinical models, providing a rationale for combination therapies to overcome chemoresistance and improve clinical outcomes in neuroblastoma patients (Ceder et al., 2021; Vernooij et al., 2021) (Figure 2)

### 3.2 Retinoblastoma (RB) cell cycle regulation

The RB tumor suppressor pathway is an essential cell cycle regulator, primarily controlling the transition from the G1 to the S phase by inhibiting E2F transcription factors. In its hypophosphorylated state, RB interacts with E2F family members and inhibits the transcription of genes required for DNA replication and cell cycle entry. Additionally, RB phosphorylation by cyclin-dependent kinases (CDKs), namely, *CDK4* and *CDK6* abrogates RB’s functional activity, leading to the release of E2F and cell cycle entry (Goel et al., 2022; Kim et al., 2022). In neuroblastoma, *RB1* gene mutations are rare; however, functional RB pathway disruption is a frequent event due to upstream oncogenic signaling, cyclin D overexpression, or *CDK4/6* hyperactivation. This functional loss promotes uncontrolled cell growth, genomic instability, and resistance to cytotoxic insults (Molenaar et al., 2008; Gogolin et al., 2013; Rader et al., 2013; De Rosa et al., 2023). There is a substantial number of experimental studies support a major role for RB pathway dysregulation. For example, gene expression profiling of high-risk neuroblastoma tumors shows the enrichment of E2F target gene signatures, suggesting RB functional loss (Wang H. et al., 2022). Emerging evidence further suggests that *MYCN* amplification cooperates closely with RB pathway dysfunction to promote uncontrolled cell cycle progression and aggressive neuroblastoma growth. *MYCN* overexpression enhances E2F-dependent transcriptional programs and promotes *CDK4/6* hyperactivation, leading to increased RB phosphorylation, accelerated G1/S phase transition, and sustained proliferative signaling. This cooperative dysregulation of *MYCN* and RB-associated pathways contributes to genomic instability, replication stress, and resistance to chemotherapy and targeted therapies in high-risk neuroblastoma.

Further, *in vitro* studies using neuroblastoma cell lines demonstrate that *CDK4* or *CDK6* overexpression increases RB phosphorylation, accelerates cell proliferation, and confers resistance to apoptosis in response to genotoxic insult. In contrast, RNAi-mediated *CDK4* or cyclin D1 knockdown reactivates RB hypophosphorylation and inhibits tumor cell proliferation (Molenaar et al., 2008; Rader et al., 2013). Moreover, PDX models show that tumors with high *CDK4/6* activity display more aggressive growth kinetics and a diminished response to chemotherapy. From a therapeutic perspective, the inhibition of *CDK4/6* has shown promise in indirectly reactivating RB tumor suppressor activity. Preclinical studies using *CDK4/6* inhibitors (such as palbociclib, ribociclib, and abemaciclib) have shown decreased RB phosphorylation, cell cycle arrest, and senescence in RB-competent neuroblastoma models (Rader et al., 2013; Rihani et al., 2015; Ferguson et al., 2025). Combination therapy studies have shown that *CDK4/6* inhibitors increase the sensitivity of neuroblastoma cells to DNA-damaging agents and radiation therapy by overcoming cell cycle-mediated DNA repair mechanisms. Early-phase translational studies also suggest that CDK4/6 inhibitors may have a role in modulating tumor immune signaling and metabolism, potentially synergizing with immunotherapy (Gogolin et al., 2013; Deng et al., 2018). Notably, the therapeutic efficacy of *CDK4/6* inhibitors seems to depend on the presence of RB protein expression, highlighting the need for biomarker-based patient selection. Ongoing clinical trials assessing the role of *CDK4/6* inhibitors in paediatric solid malignancies, including neuroblastoma, are expected to provide insight into their therapeutic utility (Rihani et al., 2015). In summary, targeting RB pathway dysregulation offers a logical approach to dampen proliferation and overcome therapy resistance in neuroblastoma (Figure 2).

### 3.3 PTEN-PI3K-AKT survival signaling

The PTEN/PI3K/AKT signaling pathway is a key regulator of cell survival, metabolism, proliferation, and angiogenesis. Phosphatase and tensin homolog (*PTEN*) are a lipid phosphatase that negatively regulates phosphoinositide 3-kinase (*PI3K*) signaling by dephosphorylating phosphatidylinositol-3,4,5-trisphosphate (*PIP3*) to phosphatidylinositol-4,5-bisphosphate (*PIP2*), thus inhibiting serine-threonine kinase (*AKT*) activation (Stambolic et al., 1998; Seront et al., 2013; Haddadi et al., 2018). The loss of *PTEN* activity results in the constitutive activation of *PI3K/AKT* signaling, which promotes tumor cell survival, apoptosis resistance, metabolic dysregulation, and pro-angiogenic signals. In many solid malignancies, *PTEN* deletion is linked to therapy resistance, immune escape, and aggressive disease, thus establishing its importance as a major tumor suppressor (Exposito et al., 2023; Malhotra et al., 2024; Rao et al., 2025). In neuroblastoma, while *PTEN* gene mutations or deletions are rare, functional *PTEN* downregulation is a common event through epigenetic and post-transcriptional regulation. Studies in clinical tumor series have shown decreased PTEN protein expression in high-risk neuroblastoma compared to low-risk neuroblastoma, which is associated with advanced disease stage and poor survival (Fransson et al., 2013). Further, methylation-specific polymerase chain reaction (PCR) and chromatin immunoprecipitation analyses have identified *PTEN* promoter hypermethylation and repressive chromatin marks as key mechanisms of gene silencing. Moreover, preclinical studies have demonstrated that regulatory microRNAs (miRNAs), such as miR-21 and members of the miR-17–92 microRNA cluster, directly inhibit *PTEN* expression in neuroblastoma cells, resulting in increased *AKT* phosphorylation and tumor cell survival (Hoebeeck et al., 2009; Kato et al., 2009; Liu et al., 2014). Emerging studies additionally indicate that *MYCN* amplification cooperates with *PTEN* suppression and activation of the PI3K-AKT-mTOR signaling axis to promote survival signaling and aggressive tumor progression in neuroblastoma. *MYCN*-driven oncogenic signaling has been associated with profound metabolic rewiring characterized by enhanced glycolysis, mitochondrial metabolic plasticity, lipid biosynthesis, and increased tolerance to oxidative stress. These metabolic adaptations enable neuroblastoma cells to survive under hypoxic and nutrient-deprived tumor microenvironment conditions while simultaneously contributing to therapeutic resistance and sustained proliferative capacity.

Additionally, functional experimental data also validate the importance of *PTEN* signaling in neuroblastoma. For instance, Restoration of *PTEN* expression decreases AKT activation, proliferation, and chemotherapy-induced apoptosis (Zhang J. et al., 2023). In xenograft models, *PTEN* expression restoration or pharmacologic inhibition of PI3K/AKT signaling suppresses tumor growth and angiogenesis. High activity of the *PI3K/AKT* pathway has also been correlated with resistance to chemotherapy and targeted therapies in relapsed neuroblastoma. From a therapeutic perspective, targeting the *PI3K/AKT* pathway is a promising approach in *PTEN*-deficient neuroblastoma (Mohlin et al., 2019; Guo et al., 2022). Early-phase clinical trials of *PI3K* inhibitors, *AKT* inhibitors, and dual *PI3K*-mammalian target of rapamycin (mTOR) inhibitors in paediatric solid tumors have shown inhibition of the biological pathway and preliminary antitumor activity. Biomarker-driven stratification based on *PTEN* expression and *PI3K* pathway activation may improve patient selection and treatment response. In summary, *PTEN* functional loss is a clinically important driver of survival signaling and therapeutic resistance in neuroblastoma (Bagatell et al., 2014; Kushner et al., 2017) (Figure 2)

### 3.4 Hippo tumor suppressor pathway

The Hippo signaling pathway is a critical tumor suppressor pathway that regulates tissue growth, organ size, stemness, and cell fate decisions via the transcriptional co-activators YAP (Yes-associated protein) and TAZ (WWTR1). In normal conditions, the upstream Hippo kinases MST1/2 and LATS1/2 phosphorylate YAP/TAZ, leading to cytoplasmic sequestration and degradation (Kumar and Hong, 2024). Inhibition or inactivation of Hippo signaling results in nuclear translocation of YAP/TAZ, allowing interaction with TEAD transcription factors and subsequent activation of gene expression programs involved in proliferation, survival, epithelial-to-mesenchymal transition (EMT), and stemness. Aberrant activation of the Hippo pathway has been increasingly linked to aggressive tumor phenotypes and resistance to therapies in various cancers, including neuroblastoma (Zanconato et al., 2016; Kim and Nam, 2025). Experimental and clinical data implicate aberrant Hippo pathway in the progression of neuroblastoma. Transcriptome profiling of high-risk neuroblastoma patients identifies a high expression signature of YAP/TAZ target genes that associate with adverse survival and increased metastatic capacity. Immunohistochemical analysis of neuroblastoma tissues shows increased nuclear YAP protein accumulation in aggressive neuroblastomas compared to low-risk neuroblastoma (Shim and Goldsmith, 2021; Mokhtari et al., 2023). *In vitro* functional studies using neuroblastoma cell lines overexpressing YAP show increased proliferation, migration, and resistance to chemotherapy-induced apoptosis. Conversely, RNAi-mediated YAP or *TAZ* gene knockdown decreases tumor sphere formation and reduces stem-like tumor cell populations, suggesting a role in tumor initiation and recurrence (Li et al., 2019; Shim and Goldsmith, 2021). Recent evidence further suggests significant functional interactions between *MYCN* signaling and Hippo/YAP/TAZ-mediated stemness pathways in neuroblastoma. *MYCN* amplification has been associated with enhanced YAP/TAZ transcriptional activity, promoting tumor stemness, survival signaling, and inhibition of neuronal differentiation programs. Moreover, combined dysregulation of *MYCN* and Hippo pathway signaling contributes to increased cellular plasticity, mesenchymal transition, metastatic potential, and the emergence of therapy-resistant neuroblastoma phenotypes characterized by aggressive tumor behavior and enhanced resistance to apoptosis. Likewise, preclinical therapeutic studies have explored approaches to inhibit YAP-TEAD transcriptional function. Small molecule inhibitors that block YAP-TEAD protein-protein interactions (PPIs) have shown decreased tumor cell viability and oncogenic transcriptional suppression in neuroblastoma and other solid tumor models. Early-phase clinical studies suggest that Hippo pathway modulation may increase chemotherapy sensitivity and reduce tumor stemness (Nguyen and Yi, 2019; Barry et al., 2021; Chen et al., 2026). While few clinical trials have focused on direct YAP inhibitors, current research efforts are directed at optimizing YAP-TEAD interaction inhibitors and testing combination therapies with chemotherapy and other targeted agents. Collectively, these observations establish aberrant Hippo pathway as a clinically important mechanism contributing to neuroblastoma aggressiveness and therapeutic resistance (Xu et al., 2025) (Figure 2)

### 3.5 DDR tumor suppressors

DDR tumor suppressor pathways are critical for maintaining genomic integrity through the coordinated regulation of DNA repair, cell cycle arrest, and apoptosis in response to genotoxic stress. The core DDR machinery includes homologous recombination DNA repair proteins, checkpoint kinases, and genome surveillance regulators. The loss or functional disruption of DDR tumor suppressors contributes to genomic instability concurrently with the development of therapeutic vulnerabilities through synthetic lethality, where tumor cells become highly reliant on secondary repair mechanisms for survival (Molinaro et al., 2021). The exploitation of these vulnerabilities has become a major focus of precision oncology in various cancers, including neuroblastoma. There is experimental evidence for the dysregulation of DDR pathways in high-risk neuroblastoma. For example, genomic profiling analyses have shown that there are genetic alterations in genes that regulate homologous recombination repair, replication stress response, and checkpoint activation (Zhao et al., 2024; Hayes et al., 2025). Functional studies in neuroblastoma cell lines have shown that homologous recombination-deficient models are highly sensitive to poly (ADP-ribose) polymerase (PARP) inhibitors. The mechanism of action of PARP inhibitors involves the inhibition of single-strand DNA repair, leading to the accumulation of double-strand breaks during replication, which are not readily repaired in homologous recombination-deficient tumor cells (Lord and Ashworth, 2017; Southgate et al., 2020). Likewise, *in vitro* studies have shown that PARP inhibition leads to DNA damage accumulation, replication fork breakdown, and apoptosis in neuroblastoma models with dysfunctional DDR signaling. PRX models have also shown a marked inhibition of tumor growth with PARP inhibitor treatment, especially in tumors with replication stress signatures (Norris et al., 2014; Colicchia et al., 2017; Durinck and Irwin, 2024; Zhao et al., 2024). *MYCN* amplification has also been strongly associated with increased oncogene-induced replication stress and selective dependence on *ATR/CHK1*-mediated checkpoint signaling and homologous recombination repair pathways for tumor cell survival. Accelerated DNA replication and transcriptional hyperactivation driven by *MYCN* contribute to replication fork instability, DNA damage accumulation, and genomic instability in high-risk neuroblastoma. Consequently, *MYCN*-amplified neuroblastoma cells exhibit synthetic lethal vulnerabilities to PARP and *ATR* inhibition, as disruption of these compensatory DDR pathways further exacerbates replication stress and promotes apoptotic cell death. Clinical and translational research is increasingly supporting the use of PARP inhibition in neuroblastoma. Early-phase clinical trials of PARP inhibitors, such as olaparib and talazoparib, in paediatric solid malignancies have shown acceptable safety profiles and evidence of biological pathway suppression. Combination clinical trials of PARP inhibitors with DNA-damaging chemotherapy or radiation therapy have shown increased tumor responses in relapsed or refractory neuroblastoma patients (Takagi et al., 2022; Pearson et al., 2023; Sun et al., 2023). Notably, biomarker studies suggest that tumors with homologous recombination deficiency signatures or high replication stress markers show increased response rates to PARP inhibition. Further, PARP inhibitor therapy represents a paradigm for tumor suppressor-directed therapy, which targets underlying DDR tumor suppressor deficiencies rather than oncogenic drivers. Ongoing clinical trials seek to define predictive biomarkers of response and to refine combination strategies (Hoppe et al., 2018; Tsilingiri et al., 2024). Taken together, targeting DDR tumor suppressor vulnerabilities through PARP inhibition is a promising approach for high-risk neuroblastoma (Figure 2).

## 4 Epigenetic tumor suppressor silencing in neuroblastoma: mechanisms and therapeutic implications

**FIGURE 3:**
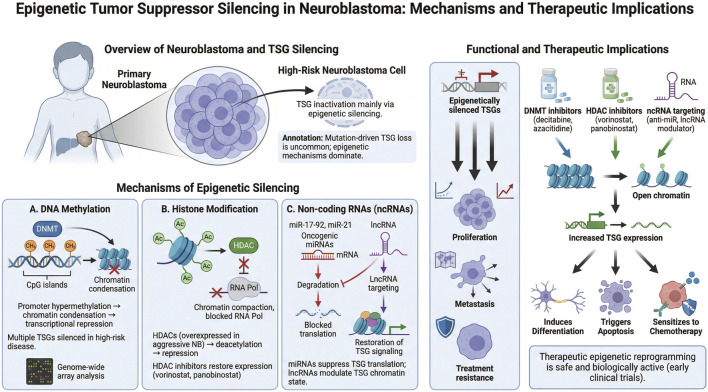
Schematic representation of epigenetic silencing mechanisms of TSGs and their targeted therapies in neuroblastoma. In high-risk neuroblastoma, the inactivation of TSG is mainly due to DNA methylation, histone deacetylation, and non-coding RNA-mediated repression. The schematic diagram above lists the various therapeutic strategies that include DNA methyltransferase (DNMT) inhibitors, histone deacetylase (HDAC) inhibitors, and non-coding RNA-targeted therapies, which work in concert to induce an open chromatin structure, enhance the expression of TSG, and induce differentiation and apoptosis, thereby making the cells more sensitive to conventional chemotherapeutic agents, thus providing a rationale for the use of epigenetic reprogramming as a promising precision medicine approach.

Epigenetic dysregulation is a major mechanism of inactivation of TSGs in neuroblastoma, especially in high-risk disease, where the traditional mutation-based loss of TSG function is relatively less common. Instead, reversible epigenetic modifications, such as DNA methylation, histone modification, and ncRNA regulation are predominant mechanisms of suppressing the transcription of genes that control cellular differentiation, apoptosis, genomic integrity, and cell cycle regulation (Domingo-Fernandez et al., 2013; Parodi et al., 2016). These epigenetic modifications have a special significance in neuroblastoma because of their dynamic and reversible nature, making them attractive targets for therapeutic intervention. For example, DNA methyltransferases mediate gene silencing through methylation of CpG-rich promoter regions, resulting in chromatin condensation and transcriptional repression (Decock et al., 2012; Jones, 2012). High-risk neuroblastoma is characterized by extensive DNA methylation abnormalities, including hypermethylation of multiple TSG promoters. Genome-wide methylation analysis using high-density methylation arrays and whole-genome bisulfite sequencing has identified robust methylation signatures that are highly associated with aggressive tumor behavior, metastatic phenotype, and resistance to therapy. Functional analysis has shown that promoter hypermethylation is responsible for the silencing of genes involved in neuronal differentiation, apoptosis, and DNA repair (Decock et al., 2015; Fetahu and Taschner-Mandl, 2021; Yu et al., 2024; Lavoro et al., 2025). *MYCN* amplification has additionally been implicated in extensive epigenetic remodeling through recruitment of chromatin remodeling complexes, histone-modifying enzymes, histone deacetylases (HDACs), and DNA methyltransferases (DNMTs) that collectively promote transcriptional repression of TSGs. Emerging evidence further suggests that *MYCN*-mediated enhancer remodeling and chromatin reprogramming contribute to widespread silencing of differentiation-associated and tumor suppressor loci, thereby sustaining aggressive neuroblastoma phenotypes characterized by enhanced proliferation, stemness, and therapeutic resistance. For example, inhibition of DNMTs using pharmacologic inhibitors, namely, decitabine and azacitidine reverses TSG expression in neuroblastoma cell lines, resulting in differentiation and apoptosis. Additionally, *in vivo* xenograft studies have further shown that DNMT inhibition reduces tumor growth and increases sensitivity to chemotherapy. Early-phase paediatric clinical trials of low-dose decitabine have demonstrated acceptable safety and biological reactivation of silenced genes, justifying further clinical development (George et al., 2010; Dai et al., 2024). Further, *MYCN* amplification has additionally been implicated in widespread epigenetic remodeling in neuroblastoma through recruitment of chromatin remodeling complexes, histone-modifying enzymes, and DNA methyltransferases that collectively promote transcriptional silencing of TSGs. Recent studies indicate that *MYCN*-mediated enhancer remodeling and chromatin reprogramming cooperate with HDAC- and DNMT-associated pathways to suppress differentiation-associated genes and maintain aggressive tumor phenotypes characterized by increased proliferation, stemness, and therapeutic resistance. In addition to DNA methylation, histone modifications are another major mechanism of TSGs regulation. HDACs facilitate chromatin compaction through the removal of acetyl groups from histone tails, thus limiting transcriptional potential. HDAC overexpression has been linked to undifferentiated tumors, enhanced proliferation, and adverse prognosis in neuroblastoma. Preclinical studies have shown that HDAC inhibitors can reactivate TSGs transcription and induce neuronal differentiation in neuroblastoma (De los Santos et al., 2007; Oehme et al., 2008; Rettig et al., 2015). HDAC inhibitors, such as vorinostat and panobinostat induce anti-proliferative and pro-apoptotic effects in cell line and xenograft models. The mechanism of HDAC inhibition involves chromatin relaxation, which allows TSG reactivation and increased sensitivity of tumor cells to DNA damage. Combination regimens that combine HDAC inhibitors with chemotherapy, retinoids, or immunotherapy have shown synergistic antitumor activity in preclinical models, while initial clinical trials in paediatric solid malignancies, including neuroblastoma, have shown manageable toxicity and evidence of early disease stabilization (Glasser et al., 2017; Hosseini et al., 2024; El Omari et al., 2025; Yuan et al., 2025). Moreover, ncRNAs, such as miRNAs and long ncRNAs (lncRNAs), add an additional layer of epigenetics through the post-transcriptional regulation of TSG expression. Further, oncogenic miRNAs can directly target tumor suppressor mRNAs, leading to degradation or translational repression. Experimental evidence suggests that the miR-17–92 cluster and miR-21 suppress tumor suppressor regulators of apoptosis and cell cycle regulation, contributing to tumor growth and survival. Similarly, lncRNAs regulate tumor suppressor pathways through chromatin remodeling, transcriptional interference, and miRNA sponging. Experimental knockdown of oncogenic lncRNAs in neuroblastoma cell models rescues tumor suppressor pathway activity, reduces proliferation, and enhances apoptosis (Mestdagh et al., 2010; Russell et al., 2015; Yu et al., 2019; Huang et al., 2022). Additionally, clinical transcriptomic analyses demonstrate that aberrant ncRNA expression profiles are associated with neuroblastoma risk stratification and survival. In summary, both preclinical and clinical evidence support the role of epigenetic silencing of tumor suppressors as a major contributing factor to neuroblastoma progression and resistance to therapy (Mestdagh et al., 2010; Rezaei et al., 2021; Martínez-Pacheco et al., 2023). In summary, DNA methylation, histone modifications, and ncRNA regulatory pathways are currently being explored in clinical trials, and combination epigenetic therapy regimens may represent a rational therapeutic strategy to rescue tumor suppressor pathway activity and enhance treatment responses in high-risk neuroblastoma (Figure 3).

## 5 Molecular interplay between tumor suppressor pathways and TME signaling in neuroblastoma

The loss of TSG function is not only a driving force for tumor cell-intrinsic oncogenic signaling but also significantly reshapes the TME, thereby influencing immune evasion, metabolic adaptation, stromal remodeling, angiogenesis, and metastatic progression in neuroblastoma. Increasing evidence suggests that dysfunction of major tumor suppressor pathways, including *Tp53*, *PTEN*, RB, *CASZ1*, and *SRCIN1*, contributes directly to the establishment of a pro-tumorigenic and immune-suppressive microenvironment that supports tumor progression and resistance to therapy (Esteban-Villarrubia et al., 2024; Nicolini and Ferrari, 2024; Wang et al., 2024; Zahraeifard et al., 2024). Importantly, single-cell transcriptomic studies reveal that neuroblastoma displays substantial intratumoral plasticity characterized by dynamic transitions between adrenergic (ADRN) and mesenchymal (MES) cellular states, each possessing distinct metabolic, immunological, and therapeutic properties. ADRN neuroblastoma cells are typically characterized by neuronal differentiation markers and proliferative signaling, whereas MES-like tumor cells display enhanced migratory capacity, stem-like features, inflammatory signaling, immune evasion, and increased resistance to chemotherapy and targeted therapies. Recent studies indicate that tumor suppressor dysfunction contributes significantly to this lineage plasticity. Loss of *Tp53* and *PTEN* signaling has been associated with increased activation of PI3K-AKT-mTOR, NF-κB, and hypoxia-associated pathways that promote mesenchymal transition, metastatic behavior, and resistance to apoptosis. In parallel, suppression of *CASZ1*, a developmental tumor suppressor involved in neuronal differentiation, has been linked to maintenance of undifferentiated and stem-like tumor states. Similarly, loss of *SRCIN1* function enhances SRC-mediated oncogenic signaling, inflammatory pathway activation, epithelial-to-mesenchymal transition-like phenotypes, and metastatic progression. These observations collectively suggest that tumor suppressor dysfunction actively drives neuroblastoma cellular plasticity and contributes to the emergence of therapy-resistant MES populations. In addition to promoting tumor plasticity, tumor suppressor dysfunction also contributes significantly to the immune-cold phenotype characteristic of high-risk neuroblastoma. Unlike many adult malignancies, neuroblastoma exhibits a relatively low tumor mutational burden and limited neoantigen generation, contributing to poor immune recognition and weak endogenous anti-tumor immune responses (Zahraeifard et al., 2024; Zhu et al., 2024). Experimental models have demonstrated that loss of *Tp53* signaling alters the expression of immune regulatory molecules, including PD-L1, major histocompatibility complex (MHC) proteins, and cytokines involved in immune cell recruitment. In neuroblastoma models, disruption of p53 signaling has been associated with reduced antigen presentation, impaired cytotoxic T-cell infiltration, and increased secretion of immunosuppressive cytokines such as transforming growth factor-beta (*TGF-β*) and interleukin-10 (*IL-10*), thereby facilitating expansion of regulatory T cells and suppressive myeloid populations (Wang et al., 2024). Clinical transcriptomic studies have similarly identified correlations between reduced *Tp53* pathway activity and immune-excluded tumor phenotypes, supporting the role of tumor suppressor dysfunction in neuroblastoma immune evasion. These immunosuppressive mechanisms have important implications for GD2-targeted immunotherapy, which remains a cornerstone of treatment for high-risk neuroblastoma. Although anti-GD2 monoclonal antibodies have significantly improved survival outcomes, many patients ultimately develop resistance or exhibit incomplete responses. Recent studies indicate that tumor suppressor dysfunction and microenvironmental remodeling may influence responsiveness to GD2-targeted therapies. For example, loss of *Tp53* and *PTEN* signaling may reduce immune cell recruitment, impair natural killer (NK) cell-mediated cytotoxicity, and increase expression of immunosuppressive cytokines within the TME, thereby limiting antibody-dependent cellular cytotoxicity. Moreover, MES-like neuroblastoma states have been associated with reduced immunogenicity and enhanced resistance to immunotherapeutic approaches. These findings suggest that restoration of tumor suppressor signaling or modulation of associated immune pathways may enhance the efficacy of GD2-targeted immunotherapy in refractory neuroblastoma.

**FIGURE 4:**
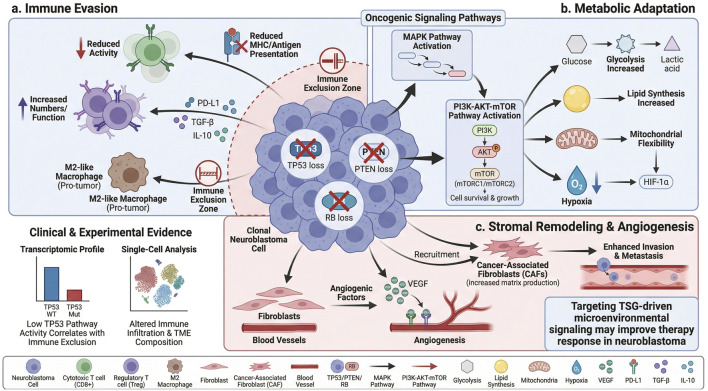
Role of TSG loss in the reprogramming of the tumor microenvironment in neuroblastoma. TSG loss of TP53, PTEN, and RB contributes to immune evasion by reducing antigen presentation, upregulating PD-L1, and secreting immunosuppressive cytokines (TGF-β, IL-10), in addition to M2 macrophage polarization. TSG dysfunction leads to the activation of MAPK and PI3K-AKT-mTOR signaling pathways, which support metabolic reprogramming by increasing glycolysis, lipogenesis, and mitochondrial plasticity in hypoxic conditions. Additionally, TSG loss contributes to stromal reorganization, the presence of cancer-associated fibroblasts, and VEGF-mediated angiogenesis.

Metabolic reprogramming represents another critical consequence of tumor suppressor dysfunction within the neuroblastoma microenvironment. Experimental metabolomic studies demonstrate that loss of *Tp53* and *PTEN* signaling promotes increased glycolysis, mitochondrial metabolic plasticity, lipogenesis, and oxidative stress tolerance through activation of PI3K-AKT-mTOR and hypoxia-inducible factor-1 alpha (*HIF-1α*) signaling pathways (Verhoeven et al., 2022; Wang L. et al., 2022; Koo et al., 2025). In neuroblastoma cell models, *PTEN* downregulation increases metabolic adaptability under hypoxic and nutrient-deprived conditions while simultaneously enhancing resistance to oxidative damage and chemotherapy-induced apoptosis. High-risk neuroblastoma tumors also display metabolic signatures associated with enhanced glycolytic flux and mitochondrial rewiring, which correlate strongly with aggressive disease behavior and therapeutic resistance (Smiles et al., 2023; Zhang W. et al., 2023). Moreover, recent studies suggest that *CASZ1* restoration suppresses pro-metastatic metabolic programs, whereas *SRCIN1* inhibits SRC-driven inflammatory and metabolic signaling pathways linked to tumor progression and stemness. Tumor suppressor dysfunction additionally exerts profound effects on stromal remodeling and extracellular matrix organization within the neuroblastoma TME. Experimental co-culture and xenograft models demonstrate that *Tp53*-deficient neuroblastoma cells secrete pro-angiogenic and pro-inflammatory mediators, including *VEGF*, *MMPs*, and cytokines that recruit CAFs and remodel extracellular matrix architecture (Qiu et al., 2024; Kimura et al., 2025). These stromal alterations contribute to enhanced tumor invasiveness, angiogenesis, metastatic dissemination, and immune exclusion. In parallel, dysfunction of *PTEN* and RB signaling pathways has been associated with increased activation of fibroblast-associated inflammatory signaling and extracellular matrix deposition. Neuroblastoma xenograft studies further demonstrate increased vascular density, stromal remodeling, and macrophage infiltration in tumors with disrupted p53 signaling (Carr-Wilkinson et al., 2010; Flesken-Nikitin et al., 2013). Importantly, M2 macrophage polarization induced by tumor suppressor dysfunction promotes secretion of immunosuppressive cytokines, angiogenic factors, and extracellular matrix remodeling proteins that further reinforce tumor progression and therapeutic resistance. Additionally, single-cell transcriptomic analyses have revealed that the disruption of p53 function influences the immune cell infiltration profile, induces the polarization of macrophages into tumor-supporting M2 macrophages, and affects interferon signaling. In the clinic, the disruption of *Tp53* signaling in tumors has been associated with a lack of response to immunotherapy and chemotherapy, highlighting the importance of *Tp53*-microenvironment interactions in tumor therapy (Wang et al., 2024; Hsiao et al., 2025; Shokouhfar et al., 2026). Collectively, these findings establish tumor suppressor dysfunction as a central regulator of neuroblastoma-specific TME reprogramming, influencing cellular plasticity, immune evasion, metabolic adaptation, stromal remodeling, and immunotherapy responsiveness. Importantly, these observations also provide strong translational rationale for therapeutic strategies integrating tumor suppressor pathway restoration with immunotherapy, metabolic targeting, stromal modulation, and precision combination therapies. Future studies integrating single-cell multi-omics profiling, spatial transcriptomics, and biomarker-guided therapeutic approaches will likely further improve understanding of neuroblastoma TME biology and facilitate development of more effective personalized therapeutic strategies (Figure 4).

## 6 Therapeutic strategies targeting tumor suppressor dysfunction

The concept of targeting the dysfunction of TSGs represents a paradigm shift in cancer therapy, especially in the context of cancers, such as neuroblastoma, where the incidence of direct oncogenic driver mutations is lower. Instead of attempting to directly compensate for the loss of tumor suppressor function, current approaches focus on the reactivation of silenced genes, the use of synthetic lethality, the application of cutting-edge gene editing tools to restore gene function, and the integration of multi-omics platforms for precision medicine (Dakal et al., 2024). There is an increasing amount of evidence to support the possibility of these approaches in neuroblastoma and other solid cancers (Table 1).

**TABLE 1:** Tumor suppressor-directed therapeutic strategies in neuroblastoma.

Therapy/Drug	Outcome	Signaling pathway affected	Target gene	Experimental model	Disease context	Evidence	References
Olaparib (PARP inhibitor)	PARP activity inhibition, partial clinical responses	DDR/HR repair	PARP1/2	Phase I paediatric clinical trial	Paediatric solid tumors incl. NB	Phase 1 clinical trials	Takagi et al. (2022)
PARP inhibitor precision therapy	Tumor regression exploiting DDR defect	DDR/HR	BARD1 mutation	Precision clinical genomics case	High-risk neuroblastoma	Translational clinical evidence	NEJM (2024)
Talazoparib + Temozolomide	Tolerated, disease stabilization	DDR + replication stress	PARP	Phase I/II paediatric trial	Paediatric solid tumors	Phase I/II clinical trial	Schafer et al. (2020)
Dinaciclib (CDK inhibitor)	Tumor growth suppression, chemo sensitization	RB/Cell cycle	CDK2/CDK9	Cell lines + orthotopic mouse + TH-MYCN	Neuroblastoma	Preclinical	Chen et al. (2016)
Palbociclib (CDK4/6 inhibitor)	G1 arrest, RB pathway restoration	RB-E2F pathway	CDK4/6 → RB	*In vitro* NB cells	Neuroblastoma	Preclinical	Rihani et al. (2015)
Decitabine (DNMT inhibitor)	TSG reactivation, apoptosis induction	Epigenetic DNA methylation	DNMT → TSG promoters	NB cell lines + xenograft	Neuroblastoma	Preclinical	Jubierre et al. (2018)
Azacitidine	Epigenetic reprogramming	DNA methylation	DNMT	Preclinical + paediatric trials	Paediatric cancers incl. NB	Preclinical	Jubierre et al. (2018)
Vorinostat (HDAC inhibitor)	Differentiation + apoptosis	Histone acetylation	HDAC → TSG chromatin	NB cell + xenograft	Neuroblastoma	Preclinical	Lautz et al. (2012)
Panobinostat	Tumor suppression + chemo synergy	Epigenetic chromatin regulation	HDAC	Preclinical NB	Neuroblastoma	Preclinical	Jubierre et al. (2018)
DNMT + HDAC Combination	Synergistic TSG reactivation	Epigenetic dual targeting	DNMT + HDAC	NB preclinical models	Neuroblastoma	Preclinical	Glasser et al. (2017)
ATR inhibitors	Replication stress targeting	DDR checkpoint	ATR	Preclinical NB	MYCN-driven NB	Preclinical	King et al. (2021), Szydzik et al. (2021)
ATM targeting	Radio sensitization	DDR signaling	ATM	Translational cancer models	Solid tumors, including NB	Translational preclinical	Durant et al. (2018)
PI3K/AKT inhibitors	Survival signaling suppression	PTEN–PI3K–AKT	PI3K/AKT	NB cell + xenograft	Neuroblastoma	Preclinical	Lang and Sandoval (2014)
mTOR inhibitors	Growth suppression	PI3K–mTOR	mTOR	Preclinical + clinical trials	NB + paediatric cancers	Preclinical	Johnsen et al. (2008)
YAP-TEAD inhibitors	Stemness + proliferation suppression	Hippo pathway	YAP/TAZ	Early preclinical	Solid tumors, including NB	Early preclinical	Feng et al. (2016)
CRISPR TSG restoration (experimental)	TSG functional recovery	Gene correction	TP53/PTEN/RB	Cell + organoid models	Experimental oncology	Early preclinical	Pandey et al. (2025)
Viral TP53 gene therapy	Tumor suppression restoration	p53 pathway	TP53	Preclinical + early clinical	Solid tumors	Preclinical	Shah et al. (2025)
Epigenome editing (dCas9)	Targeted TSG reactivation	Epigenetic	Silenced TSG promoters	Early experimental	Precision oncology	Experimental	Garcia-Bloj et al. (2016)

### 6.1 Epigenetic reactivation therapy

Epigenetic therapy aims to reactivate the expression of TSGs by targeting reversible transcriptional silencing mechanisms. DNMT inhibitors, such as decitabine and azacitidine, have shown marked efficacy in neuroblastoma models. Preclinical studies suggest that DNMT inhibitors induce the re-expression of silenced TSGs, stimulate neuronal differentiation, and increase apoptosis (Wang X. et al., 2025). In neuroblastoma xenograft models, DNMT inhibitors significantly decrease tumor mass and increase sensitivity to chemotherapeutic agents such as cisplatin and etoposide. In clinical studies, phase I trials of low-dose decitabine in relapsed and refractory solid tumors, including neuroblastoma, have been tolerable with evident biological reactivation of epigenetically silenced genes (George et al., 2010; Crabb et al., 2021). Importantly, low-dose epigenetic priming regimens seem to sensitize cancer cells to chemotherapy rather than acting as standalone agents. Further, HDAC inhibitors represent another major class of epigenetic drugs. Agents, such as vorinostat and panobinostat have shown anti-proliferative and pro-apoptotic activities in neuroblastoma cell lines and animal models (Gao et al., 2025). Mechanistically, HDAC inhibitors induce chromatin relaxation, re-activate transcription of TSGs, and increase sensitivity to DNA damage-induced apoptosis. Combination regimens that combine HDAC inhibitors with retinoic acid differentiation therapy, chemotherapy, or immunotherapy have shown synergistic antitumor activities in preclinical models (Jubierre et al., 2018; Pai et al., 2025). Early clinical trials of HDAC inhibitors in paediatric solid tumors have been manageable about toxicity and have provided initial evidence of disease stabilization. Thus, bispecific epigenetic priming therapy with DNMT and HDAC inhibitors has provided particularly encouraging preclinical data, suggesting a synergistic effect of tumor suppressor re-expression and enhanced tumor cell differentiation (Fouladi et al., 2010; Mottamal et al., 2015).

### 6.2 Synthetic lethality

Synthetic lethality takes advantage of the unique vulnerabilities of tumor cells caused by the loss of tumor suppressors. One of the most developed examples of synthetic lethality is the use of PARP inhibitors to target the defect in homologous recombination-mediated DNA repair. Preclinical studies have suggested that neuroblastoma tumors with replication stress signatures or deficiencies in DDR pathways are more sensitive to PARP inhibitors. In neuroblastoma cell lines, PARP inhibitors cause the accumulation of DNA double-strand breaks, replication fork collapse, and apoptosis (King et al., 2020; Southgate et al., 2020; Hayes et al., 2025). Additionally, preclinical studies using PDX models have shown a significant reduction in tumor growth with PARP inhibitor treatment, and this effect is more pronounced when combined with DNA-damaging chemotherapy. In clinical studies, early-phase trials of PARP inhibitors, such as olaparib and talazoparib in paediatric solid tumors shown biological efficacy and acceptable toxicity (Takagi et al., 2022; Hayes et al., 2025). Current combination studies of PARP inhibitors with chemotherapy and radiation therapy are underway. Another developing concept of synthetic lethality is the use of ATR and ATM-checkpoint pathway targeting. Preclinical studies have suggested that ATR inhibitors selectively kill tumor cells under replication stress, a common feature of MYCN-amplified neuroblastoma. Similarly, ATM inhibitors may render tumors with defects in DNA damage signaling more sensitive to chemotherapy and radiation therapy (Szydzik et al., 2021; Durinck and Irwin, 2024). Early translational studies suggest that the use of DDR pathway vulnerabilities may be a promising concept. Further, the growing recognition of MYCN-associated molecular vulnerabilities has also accelerated the development of precision therapeutic strategies targeting interconnected oncogenic signaling networks in neuroblastoma. MYCN-amplified tumors exhibit increased dependence on ATR/CHK1-mediated replication stress responses and homologous recombination repair pathways, thereby supporting the therapeutic potential of ATR inhibitors and PARP inhibitors as synthetic lethality-based approaches. In parallel, emerging epigenetic therapies targeting HDACs, BET proteins, and DNA methyltransferases have demonstrated potential to reverse MYCN-driven transcriptional and chromatin remodeling programs associated with tumor suppressor silencing. Moreover, MYCN-associated metabolic dependencies involving glycolysis, mitochondrial adaptation, and lipid biosynthesis have generated increasing interest in metabolic targeting approaches aimed at disrupting tumor bioenergetic flexibility. Importantly, growing evidence suggests that rational combination therapies simultaneously targeting replication stress pathways, epigenetic regulators, metabolic adaptation, and survival signaling networks may provide improved therapeutic efficacy while reducing the emergence of adaptive resistance mechanisms in high-risk neuroblastoma.

### 6.3 Gene restoration strategies

Gene restoration therapies are a promising and forward-looking therapeutic strategy that seeks to directly restore the function of tumor suppressors. CRISPR-mediated gene correction has provided encouraging preclinical results in cancer models. In neuroblastoma cell models, CRISPR-mediated gene correction of regulators in the tumor suppressor pathway has shown restoration of apoptotic pathways and reduction of cellular proliferation (Rabaan et al., 2023). However, the strategy is limited by issues of delivery efficacy, off-target effects, and intratumoral heterogeneity. Further, viral gene therapy approaches using adenoviral or lentiviral vectors have demonstrated the ability to restore the expression of TSGs in preclinical cancer models (Rein et al., 2006). For example, adenoviral vectors expressing TP53 have been linked to suppressed tumor growth and increased sensitivity to chemotherapy in various preclinical cancer models. Although its application in neuroblastoma is still in its infancy, viral vector-mediated gene therapy is currently under active investigation. Moreover, epigenome editing is an emerging approach that enables the targeted reactivation of silenced TSGs without modifying the underlying DNA sequence (Qadi et al., 2019; Yuan et al., 2026). CRISPR-dCas9-based epigenetic editing tools can selectively remove suppressive methylation marks or recruit transcriptional activation machinery to reactivate gene expression. Preliminary preclinical studies have shown successful reactivation of TSGs using targeted epigenome editing tools (Alves et al., 2021; Cai et al., 2023).

### 6.4 Precision multi-omics therapy selection

Precision oncology driven by multi-omics has emerged as an innovative paradigm for the management of complex malignancies characterized by the dysfunction of tumor suppressors, as seen in neuroblastoma. By providing a comprehensive understanding of tumor biology through the integration of information from various levels of biological organization, including genomics, transcriptomics, epigenomics, proteomics, and metabolomics, researchers and clinicians can achieve a comprehensive view of tumor biology (Minear et al., 2015; Rehrauer et al., 2018). This integrated approach also allows for the identification of tumor-specific molecular vulnerabilities, risk stratification, and the development of personalized therapeutic strategies. In neuroblastoma, multi-omics analysis has identified biologically distinct molecular subgroups characterized by the disruption of specific tumor suppressor pathways, unique metabolic profiles, and diverse immune microenvironment signatures, reflecting the heterogeneity of the disease and the need for personalized therapeutic strategies (Du et al., 2024; Fan et al., 2024). Preclinical multi-omics studies have shown great promise for the identification of novel therapeutic targets and the prediction of treatment outcomes. Integrated analyses of transcriptomic and proteomic data have allowed for the identification of tumor dependency on specific survival and signaling pathways, thus enabling the selection of targeted therapies that are consistent with tumor-specific biology (Guan and Quek, 2025; ElHarouni et al., 2026). Moreover, multi-omics studies have played a crucial role in the identification of synthetic lethal interactions associated with the loss of tumor suppressors, providing novel opportunities for therapeutic intervention. In terms of clinical applications, precision medicine initiatives involving multi-omics profiling have been increasingly adopted in paediatric oncology (Schäffer et al., 2024). Molecular tumor boards have been using integrated molecular data for the selection of therapeutic strategies in high-risk and relapsed neuroblastoma patients. Preliminary clinical data suggest that multi-omics-guided therapeutic decisions may improve treatment response rates while avoiding ineffective and toxic therapies. With the continued improvement of multi-omics technologies, their integration into clinical decision-making is expected to further improve clinical outcomes in patients with high-risk neuroblastoma (van Tilburg et al., 2021; Acanda De La Rocha et al., 2024).

## 7 Clinical translation challenges and future directions in targeting tumor suppressor dysfunction

Notwithstanding the major progress made in understanding the biology of TSGs, the successful translation of these findings into novel and effective therapies remains a significant challenge, particularly in the context of paediatric cancers such as neuroblastoma. The major hurdles that currently impede the successful clinical implementation of TSG-targeting strategies include intratumoral heterogeneity, clonal evolution, developmental toxicity risks, adaptive drug resistance, and the limited availability of robust paediatric clinical trial data (Gomez et al., 2022a; Han et al., 2024). Addressing these limitations will require integrated precision oncology approaches combining biomarker-guided therapy selection, multi-omics profiling, artificial intelligence (AI)-based predictive modeling, and rational combination therapeutic strategies. One of the most critical obstacles to effective clinical translation is the remarkable intratumoral heterogeneity observed in high-risk neuroblastoma (He et al., 2026; Hsu et al., 2026). Further, single-cell sequencing and spatial transcriptomic studies have demonstrated that neuroblastoma tumors contain highly diverse cellular populations with distinct adrenergic, mesenchymal, stem-like, and therapy-resistant phenotypes (Zeineldin et al., 2022). These heterogeneous tumor cell populations display substantial variability in signaling pathway activation, epigenetic states, metabolic adaptation, and therapeutic sensitivity, thereby contributing to differential treatment responses within the same tumor. Importantly, clonal evolution during treatment further promotes the emergence of resistant subclones capable of surviving selective therapeutic pressure. Recent studies suggest that chemotherapy and targeted therapies can dynamically reshape tumor cellular composition, enriching aggressive mesenchymal-like or stem-cell-associated populations characterized by enhanced plasticity, metastatic potential, immune evasion, and resistance to apoptosis (Avitabile et al., 2022; He et al., 2026). These findings emphasize the need for longitudinal molecular monitoring and adaptive therapeutic strategies capable of targeting evolving tumor ecosystems rather than static molecular alterations alone. Additionally, developmental toxicity is another major concern in paediatric neuroblastoma therapy. Many tumor suppressor pathways targeted therapeutically, including TP53, Hippo, DNMT, and HDAC signaling networks, play fundamental roles in normal embryonic development, neuronal maturation, tissue homeostasis, and organogenesis (Lu et al., 2023; ElHarouni et al., 2026). Consequently, systemic modulation of these pathways may produce unintended toxicities in rapidly developing paediatric tissues. For example, prolonged activation of p53 signaling may impair stem cell proliferation and tissue regeneration, whereas disruption of Hippo pathway signaling may interfere with organ growth and neurodevelopmental processes. Similarly, epigenetic therapies targeting DNMTs and HDACs raise substantial concerns because of their broad transcriptional effects and potential long-term consequences on chromatin organization, neuronal differentiation, endocrine regulation, and cognitive development. Although early-phase paediatric trials of DNMT and HDAC inhibitors have demonstrated manageable short-term toxicities, long-term neurodevelopmental, reproductive, and epigenomic consequences remain poorly characterized. These concerns highlight the urgent need for paediatric-specific toxicity assessment frameworks, long-term surveillance studies, and the development of more selective epigenetic modulators with reduced off-target effects. Moreover, drug resistance also remains a major clinical challenge limiting the long-term efficacy of tumor suppressor-directed therapies (Rossi et al., 2024). Although PARP inhibitors have shown significant promise in exploiting DDR vulnerabilities in neuroblastoma, acquired resistance frequently develops through multiple compensatory mechanisms. Experimental studies suggest that tumor cells may restore homologous recombination repair capacity through reactivation of BRCA-associated repair pathways, stabilization of replication forks, or upregulation of alternative DNA repair proteins, thereby diminishing PARP inhibitor sensitivity. In addition, activation of replication stress tolerance mechanisms and rewiring of ATR-CHK1 signaling pathways can promote survival under sustained genotoxic stress. Likewise, resistance to CDK4/6 inhibitors may emerge through compensatory activation of cyclin E-CDK2 signaling, RB pathway bypass mechanisms, PI3K-AKT-mTOR pathway activation, or MYCN-driven transcriptional rewiring. These adaptive signaling responses limit the durability of monotherapy approaches and strongly support the rationale for rational combination strategies targeting multiple interconnected survival pathways simultaneously.

**FIGURE 5:**
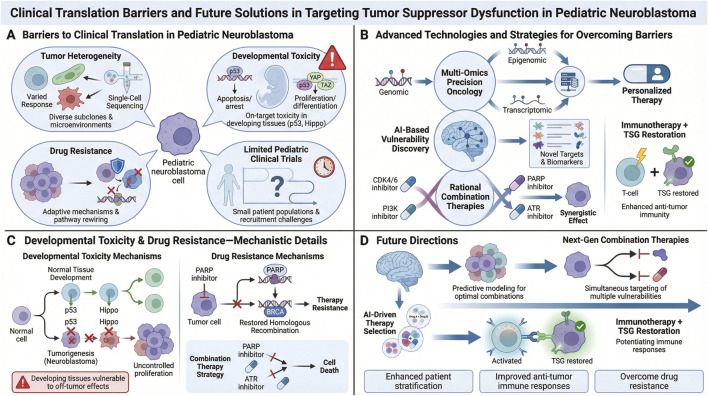
Clinical translation challenges and novel solutions for targeting tumor suppressor pathway dysfunction in paediatric neuroblastoma: The major challenges include tumor heterogeneity, developmental toxicity, resistance, and the lack of paediatric clinical trial data. The latest approaches include multi-omics precision oncology, artificial intelligence-based vulnerability prediction, combination therapies, and the combination of immunotherapy with tumor suppressor pathway restoration. The focus on mechanisms includes the risk of toxicity to developmental pathways and resistance, while the future perspective includes artificial intelligence-based therapy choice and novel approaches to combination therapies.

Another major limitation hindering clinical implementation is the restricted availability of paediatric-specific clinical trial data. Most targeted therapies are initially developed and optimized in adult malignancies before limited evaluation in paediatric populations. Consequently, many paediatric neuroblastoma trials involve relatively small patient cohorts, heterogeneous disease populations, limited follow-up durations, and insufficient long-term safety analyses (Ashique et al., 2025). Paediatric-specific pharmacokinetic, pharmacodynamic, and developmental toxicity profiles are frequently inadequately characterized, delaying therapeutic optimization for children. Additionally, the rarity and biological complexity of high-risk neuroblastoma further complicate patient recruitment, biomarker stratification, and statistical interpretation of clinical outcomes. These limitations underscore the importance of international collaborative paediatric oncology trial networks, harmonized molecular profiling strategies, and adaptive trial designs capable of rapidly integrating emerging biological insights into therapeutic decision-making. Furthermore, biomarker-guided therapeutic stratification may improve patient selection for PARP inhibitors, CDK4/6 inhibitors, epigenetic therapies, and immunomodulatory approaches while minimizing unnecessary toxicities. In parallel, AI-driven computational models are increasingly being used to predict synthetic lethal interactions, identify hidden pathway dependencies, and optimize individualized therapeutic combinations based on complex molecular datasets (Benfatto et al., 2021; Huang et al., 2025; Ing et al., 2025; Salvati et al., 2025). Early machine learning studies have already demonstrated the potential to predict drug sensitivity patterns and resistance mechanisms in paediatric tumor models with substantial accuracy. Importantly, rational combination therapies are emerging as a promising strategy to overcome compensatory resistance mechanisms and improve long-term treatment efficacy. Preclinical studies demonstrate synergistic antitumor activity when combining PARP inhibitors with ATR inhibitors, CDK4/6 inhibitors with PI3K-AKT-mTOR pathway inhibitors, or epigenetic therapies with immunotherapy and differentiation agents (Jabbour et al., 2024; Xie et al., 2024). Moreover, restoration of tumor suppressor signaling may enhance antitumor immunity by increasing antigen presentation, reversing immunosuppressive cytokine signaling, and promoting immune cell infiltration into the tumor microenvironment (Carlsen et al., 2023; Lu et al., 2025). Consequently, combining tumor suppressor-directed therapies with immune checkpoint blockade or adoptive cellular immunotherapy may represent a promising avenue for improving therapeutic responses in refractory neuroblastoma (Seliger, 2019). Current evidence supports that extensive crosstalk between MYCN, TP53, RB, PTEN/AKT, Hippo, and DDR signaling networks drive to adaptive therapeutic resistance, clonal evolution, and treatment failure in high-risk neuroblastoma. Dysregulation of these interconnected pathways promotes compensatory survival signaling, cellular plasticity, metabolic adaptation, and persistence of therapy-resistant tumor subpopulations under selective therapeutic pressure. These findings further support the development of biomarker-guided precision therapeutic strategies and rational combination therapies simultaneously targeting multiple interconnected oncogenic and tumor suppressor signaling pathways to improve treatment efficacy and overcome resistance mechanisms in neuroblastoma. In summary, future clinical translation efforts will require multidisciplinary integration of molecular oncology, paediatric pharmacology, computational biology, and translational therapeutics. Continued advances in single-cell technologies, precision medicine platforms, biomarker-guided therapy selection, and rational combination therapeutic development are expected to substantially improve the safety, efficacy, and durability of tumor suppressor-directed interventions in paediatric neuroblastoma (Figure 5).

## 8 Conclusion

TSG dysfunction represents a fundamental molecular hallmark underlying neuroblastoma progression, relapse, and therapeutic resistance. Unlike many adult malignancies that are primarily driven by high mutational burden, neuroblastoma is characterized predominantly by structural genomic alterations, epigenetic dysregulation, and disruption of regulatory signaling networks that collectively impair tumor suppressor function (Takita, 2021). Large-scale genomic and transcriptomic analyses consistently demonstrate that high-risk neuroblastoma frequently exhibits functional inactivation of key tumor suppressor pathways, including TP53, RB, PTEN, Hippo, and DDR regulators, which are strongly associated with aggressive clinical behavior and poor therapeutic responsiveness (Almeida et al., 2022). Emerging therapeutic strategies targeting tumor suppressor-associated vulnerabilities have shown encouraging translational potential in neuroblastoma. Epigenetic therapies, including DNMT and HDAC inhibitors, have demonstrated the ability to restore TSG expression, induce differentiation, and enhance chemotherapy sensitivity in preclinical neuroblastoma models (Jubierre et al., 2018). Similarly, synthetic lethality-based approaches targeting DNA repair dependencies, particularly through PARP inhibition, have shown promising activity in tumors characterized by replication stress and homologous recombination repair deficiencies (Zhao et al., 2024). In parallel, advances in genomic and transcriptomic profiling continue to facilitate identification of tumor-specific molecular vulnerabilities and biologically relevant therapeutic targets. Despite these advances, substantial challenges continue to limit successful clinical translation, including intratumoral heterogeneity, clonal evolution, developmental toxicity concerns, and adaptive resistance mechanisms. Future progress in neuroblastoma therapy will likely depend on biomarker-guided combination strategies capable of targeting interconnected tumor suppressor, metabolic, epigenetic, and immune-associated signaling networks while overcoming adaptive resistance mechanisms. Continued integration of molecular profiling technologies, translational therapeutics, and paediatric precision oncology approaches may ultimately improve therapeutic stratification and long-term outcomes for patients with high-risk neuroblastoma.

## Glossary

TSG: Tumor Suppressor Gene
ncRNAs: Non-Coding RNAs
PTMs: Post-Transcriptional Modifications
HTS: High-Throughput Sequencing
TME: Tumor Microenvironment
WGS: Whole Genome Sequencing
SNP: Single Nucleotide Polymorphism
CNTNAP2: Contactin-Associated Protein-Like 2
RNAi: RNA Interference
aCGH: Array Comparative Genomic Hybridization
DDR: DNA Damage Response
MDM2: Mouse Double Minute 2 Homolog
PDX: Patient-Derived Xenograft
RB: Retinoblastoma
CDKs: Cyclin-Dependent Kinases
PTEN: Phosphatase and Tensin Homolog
PIP3: Phosphatidylinositol-3,4,5-Trisphosphate
PIP2: Phosphatidylinositol-4,5-Bisphosphate
PI3K: Phosphoinositide 3-Kinase
AKT: Serine-Threonine Kinase
PCR: Polymerase Chain Reaction
miRNAs: MicroRNAs
mTOR: Mammalian Target of Rapamycin
YAP: Yes-Associated Protein
EMT: Epithelial-to-Mesenchymal Transition
PPIs: Protein-Protein Interactions
PARP: Poly(ADP-Ribose) Polymerase
DNMTs: DNA Methyltransferases
HDACs: Histone Deacetylases
lncRNAs: Long Non-Coding RNAs
MHC: Major Histocompatibility Complex
TGF-β: Transforming Growth Factor-Beta
IL-10: Interleukin-10
VEGF: Vascular Endothelial Growth Factor
CAFs: Cancer-Associated Fibroblasts

## References

[B1] Abu-Zaid A. Fang J. Jin H. Singh S. Pichavaram P. Wu Q. (2024). Histone lysine demethylase 4 family proteins maintain the transcriptional program and adrenergic cellular state of MYCN-amplified neuroblastoma. Cell Rep. Med. 5, 101468. 10.1016/j.xcrm.2024.101468 38508144 PMC10983111

[B2] Acanda De La Rocha A. M. Berlow N. E. Fader M. Coats E. R. Saghira C. Espinal P. S. (2024). Feasibility of functional precision medicine for guiding treatment of relapsed or refractory pediatric cancers. Nat. Med. 30, 990–1000. 10.1038/s41591-024-02848-4 38605166 PMC11031400

[B3] Almeida J. Mota I. Skoda J. Sousa E. Cidade H. Saraiva L. (2022). Deciphering the role of p53 and TAp73 in neuroblastoma: from pathogenesis to treatment. Cancers 14, 6212. 10.3390/cancers14246212 36551697 PMC9777536

[B4] Alves E. Taifour S. Dolcetti R. Chee J. Nowak A. K. Gaudieri S. (2021). Reprogramming the anti-tumor immune response via CRISPR genetic and epigenetic editing. Methods & Clin. Dev. 21, 592–606. 10.1016/j.omtm.2021.04.009 PMC8142043 34095343

[B5] An P.-G. Wu W.-J. Hu X. Zhang Z.-Q. Zhang J. (2025). Single-cell sequencing reveals tumor microenvironment features associated with the response to neoadjuvant immunochemotherapy in oral squamous cell carcinoma. Cancer Immunol. Immunother. 74, 151. 10.1007/s00262-025-04014-2 40105941 PMC11923342

[B6] Ashique S. Debnath B. Ramzan M. Taj T. Islam A. Chaudhary P. (2025). A critical review of the synergistic potential of targeting p53 and CAR T cell therapy in cancer treatment. Biomed. & Pharmacother. 189, 118308. 10.1016/j.biopha.2025.118308 40628160

[B7] Avitabile M. Bonfiglio F. Aievola V. Cantalupo S. Maiorino T. Lasorsa V. A. (2022). Single-cell transcriptomics of neuroblastoma identifies chemoresistance-associated genes and pathways. Comput. Struct. Biotechnol. J. 20, 4437–4445. 10.1016/j.csbj.2022.08.031 36051886 PMC9418686

[B8] Bagatell R. Norris R. Ingle A. M. Ahern C. Voss S. Fox E. (2014). Phase 1 trial of temsirolimus in combination with irinotecan and temozolomide in children, adolescents and young adults with relapsed or refractory solid tumors: a children’s oncology group study. Pediatr. Blood Cancer 61, 833–839. 10.1002/pbc.24874 24249672 PMC4196713

[B9] Bagci O. Tumer S. Altungoz O. (2023). Chromosome 1p status in neuroblastoma correlates with higher expression levels of miRNAs targeting neuronal differentiation pathway. Vitro Cell Dev. Biol. Anim. 59, 100–108. 10.1007/s11626-023-00750-w 36800078

[B10] Barbieri E. Mehta P. Chen Z. Zhang L. Slack A. Berg S. (2006). MDM2 inhibition sensitizes neuroblastoma to chemotherapy-induced apoptotic cell death. Mol. Cancer Ther. 5, 2358–2365. 10.1158/1535-7163.MCT-06-0305 16985070

[B11] Barford R. G. Whittle E. Weir L. Fong F. C. Goodman A. Hartley H. E. (2023). Use of optical genome mapping to detect structural variants in neuroblastoma. Cancers 15, 5233. 10.3390/cancers15215233 37958407 PMC10647738

[B12] Barry E. R. Simov V. Valtingojer I. Venier O. (2021). Recent therapeutic approaches to modulate the hippo pathway in oncology and regenerative medicine. Cells 10, 2715. 10.3390/cells10102715 34685695 PMC8534579

[B13] Benchia D. Bîcă O. D. Sârbu I. Savu B. Farcaş D. Miron I. (2025). Targeting pathways in neuroblastoma: advances in treatment strategies and clinical outcomes. Int. J. Mol. Sci. 26, 4722. 10.3390/ijms26104722 40429864 PMC12112318

[B14] Benfatto S. Serçin Ö. Dejure F. R. Abdollahi A. Zenke F. T. Mardin B. R. (2021). Uncovering cancer vulnerabilities by machine learning prediction of synthetic lethality. Mol. Cancer 20, 111. 10.1186/s12943-021-01405-8 34454516 PMC8401190

[B15] Borenäs M. Umapathy G. Lind D. E. Lai W.-Y. Guan J. Johansson J. (2024). ALK signaling primes the DNA damage response sensitizing ALK-Driven neuroblastoma to therapeutic ATR inhibition. Proc. Natl. Acad. Sci. U. S. A. 121, e2315242121. 10.1073/pnas.2315242121 38154064 PMC10769851

[B16] Cai R. Lv R. Shi X. Yang G. Jin J. (2023). CRISPR/dCas9 tools: epigenetic mechanism and application in gene transcriptional regulation. Int. J. Mol. Sci. 24, 14865. 10.3390/ijms241914865 37834313 PMC10573330

[B17] Carlsen L. Zhang S. Tian X. De La Cruz A. George A. Arnoff T. E. (2023). The role of p53 in anti-tumor immunity and response to immunotherapy. Front. Mol. Biosci. 10, 1148389. 10.3389/fmolb.2023.1148389 37602328 PMC10434531

[B18] Carr-Wilkinson J. O’Toole K. Wood K. M. Challen C. C. Baker A. G. Board J. R. (2010). High frequency of p53/MDM2/p14ARF pathway abnormalities in relapsed neuroblastoma. Clin. Cancer Res. 16, 1108–1118. 10.1158/1078-0432.CCR-09-1865 20145180 PMC2842933

[B19] Ceder S. Eriksson S. E. Liang Y. Y. Cheteh E. H. Zhang S. M. Fujihara K. M. (2021). Mutant p53-reactivating compound APR-246 synergizes with asparaginase in inducing growth suppression in acute lymphoblastic leukemia cells. Cell Death Dis. 12, 709. 10.1038/s41419-021-03988-y 34267184 PMC8282662

[B20] Chen L. Rousseau R. F. Middleton S. A. Nichols G. L. Newell D. R. Lunec J. (2015). Pre-clinical evaluation of the MDM2-p53 antagonist RG7388 alone and in combination with chemotherapy in neuroblastoma. Oncotarget 6, 10207–10221. 10.18632/oncotarget.3504 25844600 PMC4496350

[B21] Chen Z. Wang Z. Pang J. C. Yu Y. Bieerkehazhi S. Lu J. (2016). Multiple CDK inhibitor dinaciclib suppresses neuroblastoma growth via inhibiting CDK2 and CDK9 activity. Sci. Rep. 6, 29090. 10.1038/srep29090 27378523 PMC4932496

[B22] Chen K. Li H. Luo Y. Liu J. Liu H. Tian Y. (2025). SNP array analysis facilitates the identification of novel chromosomal alterations associated with disease and SNPs related to adverse drug reactions in neuroblastoma. Oncol. Lett. 29, 242. 10.3892/ol.2025.14988 40486086 PMC12142285

[B23] Chen F. Su J. Liu Y. Zhang Z. Li S. Yuan Y. (2026). Targeting YAP/TAZ–TEAD and their protein-protein interaction for precision cancer therapy. Eur. J. Med. Chem. 302, 118330. 10.1016/j.ejmech.2025.118330 41192283

[B24] Colicchia V. Petroni M. Guarguaglini G. Sardina F. Sahún-Roncero M. Carbonari M. (2017). PARP inhibitors enhance replication stress and cause mitotic catastrophe in MYCN-dependent neuroblastoma. Oncogene 36, 4682–4691. 10.1038/onc.2017.40 28394338

[B25] Crabb S. J. Danson S. Catto J. W. F. Hussain S. Chan D. Dunkley D. (2021). Phase I trial of DNA methyltransferase inhibitor guadecitabine combined with cisplatin and gemcitabine for solid malignancies including urothelial carcinoma (SPIRE). Clin. Cancer Res. 27, 1882–1892. 10.1158/1078-0432.CCR-20-3946 33472913 PMC7611191

[B28] Dai W. Qiao X. Fang Y. Guo R. Bai P. Liu S. (2024). Epigenetics-targeted drugs: current paradigms and future challenges. Sig Transduct. Target Ther. 9, 332. 10.1038/s41392-024-02039-0 PMC11627502 39592582

[B29] Dakal T. C. Dhabhai B. Pant A. Moar K. Chaudhary K. Yadav V. (2024). Oncogenes and tumor suppressor genes: functions and roles in cancers. MedComm 5, e582. 10.1002/mco2.582 38827026 PMC11141506

[B30] De los Santos M. Zambrano A. Sánchez-Pacheco A. Aranda A. (2007). Histone deacetylase inhibitors regulate retinoic acid receptor β expression in neuroblastoma cells by both transcriptional and posttranscriptional mechanisms. Mol. Endocrinol. 21, 2416–2426. 10.1210/me.2007-0151 17622583

[B31] De Rosa P. Severi F. Zadran S. K. Russo M. Aloisi S. Rigamonti A. (2023). MYCN amplification, along with wild-type RB1 expression, enhances CDK4/6 inhibitors’ efficacy in neuroblastoma cells. Int. J. Mol. Sci. 24, 5408. 10.3390/ijms24065408 36982482 PMC10049239

[B32] Decock A. Ongenaert M. Hoebeeck J. De Preter K. Van Peer G. Van Criekinge W. (2012). Genome-wide promoter methylation analysis in neuroblastoma identifies prognostic methylation biomarkers. Genome Biol. 13, R95. 10.1186/gb-2012-13-10-r95 23034519 PMC3491423

[B33] Decock A. Ongenaert M. Cannoodt R. Verniers K. Wilde B. D. Laureys G. (2015). Methyl-CpG-binding domain sequencing reveals a prognostic methylation signature in neuroblastoma. Oncotarget 7, 1960–1972. 10.18632/oncotarget.6477 PMC4811509 26646589

[B34] Deng J. Wang E. S. Jenkins R. W. Li S. Dries R. Yates K. (2018). CDK4/6 inhibition augments antitumor immunity by enhancing T-cell activation. Cancer Discov. 8, 216–233. 10.1158/2159-8290.CD-17-0915 29101163 PMC5809273

[B35] Domingo-Fernandez R. Watters K. Piskareva O. Stallings R. L. Bray I. (2013). The role of genetic and epigenetic alterations in neuroblastoma disease pathogenesis. Pediatr. Surg. Int. 29, 101–119. 10.1007/s00383-012-3239-7 23274701 PMC3557462

[B36] Dong Y. Xia W. Yang Z. Ren L. Wang H. Zhou Y. (2026). Structural variation drives enhancer hijacking via 3D genome disruption in ccRCC. Npj Digit. Med. 9, 85. 10.1038/s41746-025-02186-w 41495388 PMC12852824

[B37] Du B. Zhang Y. Zhang P. Zhang M. Yu Z. Li L. (2024). Joint metabolomics and transcriptomics analysis systematically reveal the impact of MYCN in neuroblastoma. Sci. Rep. 14, 20155. 10.1038/s41598-024-71211-x 39215128 PMC11364762

[B38] Durant S. T. Zheng L. Wang Y. Chen K. Zhang L. Zhang T. (2018). The brain-penetrant clinical ATM inhibitor AZD1390 radiosensitizes and improves survival of preclinical brain tumor models. Sci. Adv. 4, eaat1719. 10.1126/sciadv.aat1719 29938225 PMC6010333

[B39] Durinck K. Irwin M. S. (2024). DNA repair and replicative stress addiction in neuroblastoma. EJC Paediatr. Oncol. 4, 100177. 10.1016/j.ejcped.2024.100177

[B40] El Omari N. Bakrim S. Elhrech H. Aanniz T. Balahbib A. Lee L.-H. (2025). Clinical efficacy and mechanistic insights of FDA-approved HDAC inhibitors in the treatment of lymphoma. Eur. J. Pharm. Sci. 208, 107057. 10.1016/j.ejps.2025.107057 40043823

[B41] ElHarouni D. Hernansaiz-Ballesteros R. Peterziel H. Balasubramanian G. P. Previti C. Schramm K. (2026). Integrative multiomics and drug sensitivity profiling reveal potential biomarkers and therapeutic strategies in pediatric solid tumors. Cancer Res. 86, 773–784. 10.1158/0008-5472.CAN-24-1938 41417259 PMC12862342

[B42] Esteban-Villarrubia J. Ballesteros P. A. Martín-Serrano M. Vico M. R. Funes J. M. Velasco G. de (2024). Mechanisms of immune evasion in PTEN loss prostate cancer. Immuno 4, 444–460. 10.3390/immuno4040028

[B43] Exposito F. Redrado M. Houry M. Hastings K. Molero-Abraham M. Lozano T. (2023). PTEN loss confers resistance to Anti-PD-1 therapy in non-small cell lung cancer by increasing tumor infiltration of regulatory T cells. Cancer Res. 83, 2513–2526. 10.1158/0008-5472.CAN-22-3023 37311042

[B44] Fan J. Tang S. Kong X. Cun Y. (2024). Integrating multi-omics data reveals neuroblastoma subtypes in the tumor microenvironment. Life Sci. 359, 123236. 10.1016/j.lfs.2024.123236 39532261

[B45] Feng J. Gou J. Jia J. Yi T. Cui T. Li Z. (2016). Verteporfin, a suppressor of YAP–TEAD complex, presents promising antitumor properties on ovarian cancer. Onco Targets Ther. 9, 5371–5381. 10.2147/OTT.S109979 27621651 PMC5010158

[B46] Ferguson K. M. Abou Grealy F. M. Y. Philpott A. (2025). CDK4/6 inhibitors display a class effect in inducing differentiation of neuroblastoma cells. Wellcome Open Res. 9, 667. 10.12688/wellcomeopenres.23190.2 41146951 PMC12553970

[B47] Fetahu I. S. Taschner-Mandl S. (2021). Neuroblastoma and the epigenome. Cancer Metastasis Rev. 40, 173–189. 10.1007/s10555-020-09946-y 33404859 PMC7897201

[B48] Flesken-Nikitin A. Hwang C.-I. Cheng C.-Y. Michurina T. V. Enikolopov G. Nikitin A. Y. (2013). Ovarian surface epithelium at the junction area contains a cancer-prone stem cell niche. Nature 495, 241–245. 10.1038/nature11979 23467088 PMC3982379

[B49] Fouladi M. Park J. R. Stewart C. F. Gilbertson R. J. Schaiquevich P. Sun J. (2010). Pediatric phase I trial and pharmacokinetic study of vorinostat: a children’s oncology group phase I consortium report. J. Clin. Oncol. 28, 3623–3629. 10.1200/JCO.2009.25.9119 20606092 PMC2917318

[B50] Fransson S. Kogner P. Martinsson T. Ejeskär K. (2013). Aggressive neuroblastomas have high p110alpha but low p110delta and p55alpha/p50alpha protein levels compared to low stage neuroblastomas. J. Mol. Signal 8, 4. 10.1186/1750-2187-8-4 23597230 PMC3639884

[B51] Fransson S. Martinez-Monleon A. Johansson M. Sjöberg R.-M. Björklund C. Ljungman G. (2020). Whole-genome sequencing of recurrent neuroblastoma reveals somatic mutations that affect key players in cancer progression and telomere maintenance. Sci. Rep. 10, 22432. 10.1038/s41598-020-78370-7 33384420 PMC7775426

[B52] Fujita T. Igarashi J. Okawa E. R. Gotoh T. Manne J. Kolla V. (2008). CHD5, a tumor suppressor gene deleted from 1p36.31 in neuroblastomas. J. Natl. Cancer Inst. 100, 940–949. 10.1093/jnci/djn176 18577749 PMC2483574

[B53] Gamble L. D. Kees U. R. Tweddle D. A. Lunec J. (2012). MYCN sensitizes neuroblastoma to the MDM2-p53 antagonists Nutlin-3 and MI-63. Oncogene 31, 752–763. 10.1038/onc.2011.270 21725357 PMC3191119

[B54] Gao T. Fu S. Quan X. Sun J. Jiang M. Li J. (2025). Advancing epigenetic combination therapy in oncology: multifunctional nano-drug delivery systems for synergistic efficacy and precision modulation. IJN 20, 14853–14883. 10.2147/IJN.S566173 41404378 PMC12704357

[B55] Garcia-Bloj B. Moses C. Sgro A. Plani-Lam J. Arooj M. Duffy C. (2016). Waking up dormant tumor suppressor genes with zinc fingers, TALEs and the CRISPR/dCas9 system. Oncotarget 7, 60535–60554. 10.18632/oncotarget.11142 27528034 PMC5312401

[B56] George R. E. Lahti J. M. Adamson P. C. Zhu K. Finkelstein D. Ingle A. M. (2010). Phase I study of decitabine with doxorubicin and cyclophosphamide in children with neuroblastoma and other solid tumors: a children’s oncology group study. Pediatr. Blood Cancer 55, 629–638. 10.1002/pbc.22607 20589651 PMC3025700

[B57] Gherardi S. Valli E. Erriquez D. Perini G. (2013). MYCN-Mediated transcriptional repression in neuroblastoma: the other side of the coin. Front. Oncol. 3, 42. 10.3389/fonc.2013.00042 23482921 PMC3593680

[B58] Glasser C. L. Lee A. Eslin D. Marks L. Modak S. Glade Bender J. L. (2017). Epigenetic combination therapy for children with secondary myelodysplastic syndrome (MDS)/acute myeloid leukemia (AML) and concurrent solid tumor relapse. J. Pediatr. Hematol. Oncol. 39, 560–564. 10.1097/MPH.0000000000000868 28562519 PMC5708164

[B59] Goel S. Bergholz J. S. Zhao J. J. (2022). Targeting cyclin-dependent kinases 4 and 6 in cancer. Nat. Rev. Cancer 22, 356–372. 10.1038/s41568-022-00456-3 35304604 PMC9149100

[B60] Gogolin S. Ehemann V. Becker G. Brueckner L. M. Dreidax D. Bannert S. (2013). CDK4 inhibition restores G_1_-S arrest in MYCN-amplified neuroblastoma cells in the context of doxorubicin-induced DNA damage. Cell Cycle 12, 1091–1104. 10.4161/cc.24091 23462184 PMC3646865

[B61] Gomez R. L. Ibragimova S. Ramachandran R. Philpott A. Ali F. R. (2022a). Tumoral heterogeneity in neuroblastoma. Biochim. Biophys. Acta Rev. Cancer 1877, 188805. 10.1016/j.bbcan.2022.188805 36162542

[B62] Gomez R. L. Woods L. M. Ramachandran R. Abou Tayoun A. N. Philpott A. Ali F. R. (2022b). Super-enhancer associated core regulatory circuits mediate susceptibility to retinoic acid in neuroblastoma cells. Front. Cell Dev. Biol. 10, 943924. 10.3389/fcell.2022.943924 36147741 PMC9485839

[B63] Grossmann L. D. Chen C.-H. Uzun Y. Thadi A. Wolpaw A. J. Louault K. (2024). Identification and characterization of chemotherapy-resistant high-risk neuroblastoma persister cells. Cancer Discov. 14, 2387–2406. 10.1158/2159-8290.CD-24-0046 39083807 PMC11609622

[B64] Guan A. Quek C. (2025). Single-cell multi-omics: insights into therapeutic innovations to advance treatment in cancer. Int. J. Mol. Sci. 26, 2447. 10.3390/ijms26062447 40141092 PMC11942442

[B65] Gundem G. Levine M. F. Roberts S. S. Cheung I. Y. Medina-Martínez J. S. Feng Y. (2023). Clonal evolution during metastatic spread in high-risk neuroblastoma. Nat. Genet. 55, 1022–1033. 10.1038/s41588-023-01395-x 37169874 PMC11481711

[B66] Guo Y. Guo D. Zhang S. Zhang Y. He X. Jiang X. (2022). Inhibition of PI3 kinase isoform p110α suppresses neuroblastoma growth and induces the reduction of anaplastic lymphoma kinase. Cell Biosci. 12, 210. 10.1186/s13578-022-00946-9 36585695 PMC9801621

[B68] Haddadi N. Lin Y. Travis G. Simpson A. M. Nassif N. T. McGowan E. M. (2018). PTEN/PTENP1: “regulating the regulator of RTK-dependent PI3K/Akt signalling”. New Targets Cancer Therapy. Mol Cancer 17, 37. 10.1186/s12943-018-0803-3 29455665 PMC5817727

[B69] Han M. Niu H. Duan F. Wang Z. Zhang Z. Ren H. (2024). Research status and development trends of omics in neuroblastoma a bibliometric and visualization analysis. Front. Oncol. 14, 1383805. 10.3389/fonc.2024.1383805 39450262 PMC11499224

[B70] Hayes M. N. Cohen-Gogo S. Kee L. Xiong X. Weiss A. Layeghifard M. (2025). DNA damage response deficiency enhances neuroblastoma progression and sensitivity to combination PARP and ATR inhibition. Cell Rep. 44, 115537. 10.1016/j.celrep.2025.115537 40220294

[B71] He G. He S. Jing X. Dai Y. Guo X. Gao J. (2026). Dissecting neuroblastoma heterogeneity through single-cell multi-omics: insights into development, immunity, and therapeutic resistance. Oncogene 45, 123–139. 10.1038/s41388-025-03635-2 41309932 PMC12738292

[B72] Hoebeeck J. Michels E. Pattyn F. Combaret V. Vermeulen J. Yigit N. (2009). Aberrant methylation of candidate tumor suppressor genes in neuroblastoma. Cancer Lett. 273, 336–346. 10.1016/j.canlet.2008.08.019 18819746

[B73] Hoppe M. M. Sundar R. Tan D. S. P. Jeyasekharan A. D. (2018). Biomarkers for homologous recombination deficiency in cancer. J. Natl. Cancer Inst. 110, 704–713. 10.1093/jnci/djy085 29788099

[B74] Hosseini M.-S. Sanaat Z. Akbarzadeh M. A. Vaez-Gharamaleki Y. Akbarzadeh M. (2024). Histone deacetylase inhibitors for leukemia treatment: current status and future directions. Eur. J. Med. Res. 29, 514. 10.1186/s40001-024-02108-8 39456044 PMC11515273

[B75] Hsiao Y.-J. Hsieh M.-S. Chang G.-C. Hsu Y.-C. Wang C.-Y. Chen Y.-M. (2025). Tp53 determines the spatial dynamics of M1/M2 tumor-associated macrophages and M1-driven tumoricidal effects. Cell Death Dis. 16, 38. 10.1038/s41419-025-07346-0 39843434 PMC11754596

[B76] Hsu C.-Y. Askar S. Alshkarchy S. S. Nayak P. P. Attabi K. A. L. Khan M. A. (2026). AI-driven multi-omics integration in precision oncology: bridging the data deluge to clinical decisions. Clin. Exp. Med. 26, 29. 10.1007/s10238-025-01965-9 PMC12634751 41266662

[B77] Hu X. Zheng W. Zhu Q. Gu L. Du Y. Han Z. (2019). Increase in DNA damage by MYCN knockdown through regulating nucleosome organization and chromatin state in neuroblastoma. Front. Genet. 10, 684. 10.3389/fgene.2019.00684 31396265 PMC6667652

[B78] Huang S. Gong N. Li J. Hong M. Li L. Zhang L. (2022). The role of ncRNAs in neuroblastoma: mechanisms, biomarkers and therapeutic targets. Biomark. Res. 10, 18. 10.1186/s40364-022-00368-2 35392988 PMC8991791

[B79] Huang X. Huang S. Reina C. Šabanović B. Roberto M. Aicher A. (2025). Single-cell multi-omics and machine learning for dissecting stemness in cancer. Brief. Bioinform 26, bbaf566. 10.1093/bib/bbaf566 41159730 PMC12570030

[B80] Ing A. Andrades A. Cosenza M. R. Korbel J. O. (2025). Integrating multimodal cancer data using deep latent variable path modelling. Nat. Mach. Intell. 7, 1053–1075. 10.1038/s42256-025-01052-4 40709098 PMC12283373

[B82] Geng J. Wang X. Zhao L. Zhang J. Niu H. (2024). Segmental chromosome aberrations as a prognostic factor of neuroblastoma: a meta-analysis and systematic review. Transl. Pediatrics 13. 10.21037/tp-24-200 PMC11543117 39524401

[B83] Jabbour S. K. Kumar R. Anderson B. Chino J. P. Jethwa K. R. McDowell L. (2024). Combinatorial approaches for chemotherapies and targeted therapies with radiation: united efforts to innovate in patient care. Int. J. Radiat. Oncology*Biology*Physics 118, 1240–1261. 10.1016/j.ijrobp.2024.01.010 38216094

[B84] Janoueix-Lerosey I. Schleiermacher G. Michels E. Mosseri V. Ribeiro A. Lequin D. (2009). Overall genomic pattern is a predictor of outcome in neuroblastoma. J. Clin. Oncol. 27, 1026–1033. 10.1200/JCO.2008.16.0630 19171713

[B85] Johnsen J. I. Segerström L. Orrego A. Elfman L. Henriksson M. Kågedal B. (2008). Inhibitors of Mammalian target of rapamycin downregulate MYCN protein expression and inhibit neuroblastoma growth *in vitro* and *in vivo* . Oncogene 27, 2910–2922. 10.1038/sj.onc.1210938 18026138

[B86] Jones P. A. (2012). Functions of DNA methylation: islands, start sites, gene bodies and beyond. Nat. Rev. Genet. 13, 484–492. 10.1038/nrg3230 22641018

[B87] Jubierre L. Jiménez C. Rovira E. Soriano A. Sábado C. Gros L. (2018). Targeting of epigenetic regulators in neuroblastoma. Exp. Mol. Med. 50, 51. 10.1038/s12276-018-0077-2 29700278 PMC5938021

[B88] Kato M. Putta S. Wang M. Yuan H. Lanting L. Nair I. (2009). TGF-β activates akt kinase through a microRNA-dependent amplifying circuit targeting PTEN. Nat. Cell Biol. 11, 881–889. 10.1038/ncb1897 19543271 PMC2744130

[B89] Katta S. S. Nagati V. Paturi A. S. V. Murakonda S. P. Murakonda A. B. Pandey M. K. (2023). Neuroblastoma: emerging trends in pathogenesis, diagnosis, and therapeutic targets. J. Control. Release 357, 444–459. 10.1016/j.jconrel.2023.04.001 37023798

[B90] Keane S. de Weerd H. A. Ejeskär K. (2022). DLG2 impairs dsDNA break repair and maintains genome integrity in neuroblastoma. DNA Repair (Amst) 112, 103302. 10.1016/j.dnarep.2022.103302 35217496

[B91] Keshelava N. Zuo J. J. Chen P. Waidyaratne S. N. Luna M. C. Gomer C. J. (2001). Loss of p53 function confers high-level multidrug resistance in neuroblastoma cell lines. Cancer Res. 61, 6185–6193. 11507071

[B92] Kim H.-S. Nam J.-S. (2025). The multifaceted role of YAP in the tumor microenvironment and its therapeutic implications in cancer. Exp. Mol. Med. 57, 2201–2213. 10.1038/s12276-025-01551-9 41028521 PMC12586452

[B93] Kim S. Leong A. Kim M. Yang H. W. (2022). CDK4/6 initiates Rb inactivation and CDK2 activity coordinates cell-cycle commitment and G1/S transition. Sci. Rep. 12, 16810. 10.1038/s41598-022-20769-5 36207346 PMC9546874

[B94] Kimura E. Hayashi Y. Nakagawa K. Saiki H. Kato M. Uema R. (2025). p53 deficiency in Colon cancer cells promotes tumor progression through the modulation of meflin in fibroblasts. Cancer Sci. 116, 1871–1882. 10.1111/cas.70026 40241262 PMC12210049

[B95] King D. Li X. D. Almeida G. S. Kwok C. Gravells P. Harrison D. (2020). MYCN expression induces replication stress and sensitivity to PARP inhibition in neuroblastoma. Oncotarget 11, 2141–2159. 10.18632/oncotarget.27329 32577161 PMC7289530

[B96] King D. Southgate H. E. D. Roetschke S. Gravells P. Fields L. Watson J. B. (2021). Increased replication stress determines ATR inhibitor sensitivity in neuroblastoma cells. Cancers (Basel) 13, 6215. 10.3390/cancers13246215 34944835 PMC8699051

[B97] Kiss N. B. Kogner P. Johnsen J. I. Martinsson T. Larsson C. Geli J. (2012). Quantitative global and gene-specific promoter methylation in relation to biological properties of neuroblastomas. BMC Med. Genet. 13, 83. 10.1186/1471-2350-13-83 22984959 PMC3495052

[B98] Koo K. Y. Moon K. Song H. S. Lee M.-S. (2025). Metabolic regulation by p53: implications for cancer therapy. Mol. Cells 48, 100198. 10.1016/j.mocell.2025.100198 39986611 PMC11925517

[B99] Kumar R. Hong W. (2024). Hippo signaling at the hallmarks of cancer and drug resistance. Cells 13, 564. 10.3390/cells13070564 38607003 PMC11011035

[B100] Kushner B. H. Cheung N.-K. V. Modak S. Becher O. J. Basu E. M. Roberts S. S. (2017). A phase I/Ib trial targeting the Pi3k/Akt pathway using perifosine: long-term progression-free survival of patients with resistant neuroblastoma. Int. J. Cancer 140, 480–484. 10.1002/ijc.30440 27649927 PMC5118186

[B101] Lakoma A. Barbieri E. Agarwal S. Jackson J. Chen Z. Kim Y. (2015). The MDM2 small-molecule inhibitor RG7388 leads to potent tumor inhibition in p53 wild-type neuroblastoma. Cell Death Discov. 1, 15026. 10.1038/cddiscovery.2015.26 26998348 PMC4794278

[B102] Lang W. H. Sandoval J. A. (2014). Detection of PI3K inhibition in human neuroblastoma using multiplex luminex bead immunoassay: a targeted approach for pathway analysis. SLAS Discov. 19, 1235–1245. 10.1177/1087057114545650 25092063

[B103] Lautz T. B. Jie C. Clark S. Naiditch J. A. Jafari N. Qiu Y.-Y. (2012). The effect of vorinostat on the development of resistance to doxorubicin in neuroblastoma. PLoS One 7, e40816. 10.1371/journal.pone.0040816 22829886 PMC3400660

[B104] Lavoro A. Ricci D. Gattuso G. Longo F. Spoto G. Vitale A. C. V. (2025). Recent advances on gene-related DNA methylation in cancer diagnosis, prognosis, and treatment: a clinical perspective. Clin. Epigenet 17, 76. 10.1186/s13148-025-01884-2 PMC12054201 40325471

[B105] Lázcoz P. Muñoz J. Nistal M. Pestaña A. Encío I. Castresana J. S. (2006). Frequent promoter hypermethylation of RASSF1A and CASP8 in neuroblastoma. BMC Cancer 6, 254. 10.1186/1471-2407-6-254 17064406 PMC1634754

[B106] Li J. Li Z. Wu Y. Wang Y. Wang D. Zhang W. (2019). The hippo effector TAZ promotes cancer stemness by transcriptional activation of SOX2 in head neck squamous cell carcinoma. Cell Death Dis. 10, 603. 10.1038/s41419-019-1838-0 31399556 PMC6689034

[B107] Li X. Cai Z. Ding S. Gao J. Wang Y. Wu Y. (2025). Integrated single-cell and machine learning analysis identifies PMAIP1 as a novel biomarker for predicting prognosis and immunotherapy response in colorectal cancer. Sci. Rep. 16, 3685. 10.1038/s41598-025-32933-8 41436831 PMC12852111

[B108] Liu Z. Yang X. Li Z. McMahon C. Sizer C. Barenboim-Stapleton L. (2011). CASZ1, a candidate tumor-suppressor gene, suppresses neuroblastoma tumor growth through reprogramming gene expression. Cell Death Differ. 18, 1174–1183. 10.1038/cdd.2010.187 21252912 PMC3131958

[B109] Liu H. Huang X. Liu X. Xiao S. Zhang Y. Xiang T. (2014). miR-21 promotes human nucleus pulposus cell proliferation through PTEN/AKT signaling. Int. J. Mol. Sci. 15, 4007–4018. 10.3390/ijms15034007 24603539 PMC3975380

[B110] Liu Z. Zhang X. Xu M. Lei H. Shern J. F. Thiele C. J. (2022). Loss of CASZ1 tumor suppressor linked to oncogenic subversion of neuroblastoma core regulatory circuitry. Cell Death Dis. 13, 871. 10.1038/s41419-022-05314-6 36243768 PMC9569368

[B111] Liu Y. Jiang N. Chen W. Zhang W. Shen X. Jia B. (2024). TRIM59-mediated ferroptosis enhances neuroblastoma development and chemosensitivity through p53 ubiquitination and degradation. Heliyon 10, e26014. 10.1016/j.heliyon.2024.e26014 38434050 PMC10906161

[B112] Liu Y. Zhao J. Wang K. Jin Y. Gong B. Gao M. (2026). Third-generation whole-genome sequencing reveals the role of CNTNAP2 as a tumor suppressor gene in high-risk neuroblastomas. J. Transl. Med. 24, 135. 10.1186/s12967-025-07671-0 41495714 PMC12870515

[B113] Lopez G. Conkrite K. L. Doepner M. Rathi K. S. Modi A. Vaksman Z. (2020). Somatic structural variation targets neurodevelopmental genes and identifies SHANK2 as a tumor suppressor in neuroblastoma. Genome Res. 30, 1228–1242. 10.1101/gr.252106.119 32796005 PMC7545140

[B114] Lord C. J. Ashworth A. (2017). PARP inhibitors: synthetic lethality in the clinic. Science 355, 1152–1158. 10.1126/science.aam7344 28302823 PMC6175050

[B115] Lu Y. Wu M. Xu Y. Yu L. (2023). The development of p53-Targeted therapies for human cancers. Cancers (Basel) 15, 3560. 10.3390/cancers15143560 37509223 PMC10377496

[B116] Lu N. Rong G. Han W. (2025). Epi-immunotherapy in cancer treatment: mechanisms, clinical progress, and future directions. Cancer Innov. 4, e70023. 10.1002/cai2.70023 41019358 PMC12460071

[B117] Mabe N. W. Huang M. Dalton G. N. Alexe G. Schaefer D. A. Geraghty A. C. (2022). Transition to a mesenchymal state in neuroblastoma confers resistance to anti-GD2 antibody via reduced expression of ST8SIA1. Nat. Cancer 3, 976–993. 10.1038/s43018-022-00405-x 35817829 PMC10071839

[B118] Malhotra J. De S. Nguyen K. Lee P. Villaflor V. (2024). Genomic and molecular alterations associated with primary resistance to immune checkpoint inhibitors. Cancer Immunol. Immunother. 73, 234. 10.1007/s00262-024-03825-z 39271499 PMC11399531

[B119] Mandriota S. J. Valentijn L. J. Lesne L. Betts D. R. Marino D. Boudal-Khoshbeen M. (2015). Ataxia-telangiectasia mutated (ATM) silencing promotes neuroblastoma progression through a MYCN independent mechanism. Oncotarget 6, 18558–18576. 10.18632/oncotarget.4061 26053094 PMC4621910

[B120] Martínez-Pacheco M. L. Hernández-Lemus E. Mejía C. (2023). Analysis of high-risk neuroblastoma transcriptome reveals gene Co-Expression signatures and functional features. Biol. (Basel) 12, 1230. 10.3390/biology12091230 PMC10525871 37759629

[B121] Mestdagh P. Boström A.-K. Impens F. Fredlund E. Van Peer G. De Antonellis P. (2010). The miR-17-92 microRNA cluster regulates multiple components of the TGFβ-pathway in neuroblastoma. Mol. Cell 40, 762–773. 10.1016/j.molcel.2010.11.038 21145484 PMC3032380

[B122] Michalowski M. B. de Fraipont F. Plantaz D. Michelland S. Combaret V. Favrot M.-C. (2008). Methylation of tumor-suppressor genes in neuroblastoma: the RASSF1A gene is almost always methylated in primary tumors. Pediatr. Blood Cancer 50, 29–32. 10.1002/pbc.21279 17570703

[B123] Minear M. A. Alessi S. Allyse M. Michie M. Chandrasekharan S. (2015). Noninvasive prenatal genetic testing: current and emerging ethical, legal, and social issues. Annu. Rev. Genomics Hum. Genet. 16, 369–398. 10.1146/annurev-genom-090314-050000 26322648

[B124] Mohlin S. Hansson K. Radke K. Martinez S. Blanco‐Apiricio C. Garcia‐Ruiz C. (2019). Anti‐tumor effects of PIM/PI3K/mTOR triple kinase inhibitor IBL‐302 in neuroblastoma. EMBO Mol. Med. 11, EMMM201810058. 10.15252/emmm.201810058 31310053 PMC6685085

[B125] Mokhtari R. B. Ashayeri N. Baghaie L. Sambi M. Satari K. Baluch N. (2023). The hippo pathway effectors YAP/TAZ-TEAD oncoproteins as emerging therapeutic targets in the tumor microenvironment. Cancers 15, 3468. 10.3390/cancers15133468 37444578 PMC10340833

[B126] Molenaar J. J. Ebus M. E. Koster J. van Sluis P. van Noesel C. J. M. Versteeg R. (2008). Cyclin D1 and CDK4 activity contribute to the undifferentiated phenotype in neuroblastoma. Cancer Res. 68, 2599–2609. 10.1158/0008-5472.CAN-07-5032 18413728

[B127] Molenaar J. J. Koster J. Zwijnenburg D. A. van Sluis P. Valentijn L. J. van der Ploeg I. (2012). Sequencing of neuroblastoma identifies chromothripsis and defects in neuritogenesis genes. Nature 483, 589–593. 10.1038/nature10910 22367537

[B128] Molinaro C. Martoriati A. Cailliau K. (2021). Proteins from the DNA damage response: regulation, dysfunction, and anticancer strategies. Cancers 13, 3819. 10.3390/cancers13153819 34359720 PMC8345162

[B129] Montella A. Tirelli M. Lasorsa V. A. Aievola V. Cerbone V. Manganiello R. (2025). Regulatory non-coding somatic mutations as drivers of neuroblastoma. Br. J. Cancer 132, 469–480. 10.1038/s41416-025-02939-0 39843641 PMC11876587

[B130] Mottamal M. Zheng S. Huang T. L. Wang G. (2015). Histone deacetylase inhibitors in clinical studies as templates for new anticancer agents. Molecules 20, 3898–3941. 10.3390/molecules20033898 25738536 PMC4372801

[B131] NEJM (2024). Response to PARP inhibition in BARD1-Mutated refractory neuroblastoma. N. Engl. J. Med. 391, 1464. 10.1056/NEJMx240007 39413393

[B132] Nguyen C. Yi C. (2019). YAP/TAZ signaling and resistance to cancer therapy. Trends Cancer 5, 283–296. 10.1016/j.trecan.2019.02.010 31174841 PMC6557283

[B133] Nicolini A. Ferrari P. (2024). Involvement of tumor immune microenvironment metabolic reprogramming in colorectal cancer progression, immune escape, and response to immunotherapy. Front. Immunol. 15, 1353787. 10.3389/fimmu.2024.1353787 39119332 PMC11306065

[B134] Norris R. E. Adamson P. C. Nguyen V. T. Fox E. (2014). Preclinical evaluation of the PARP inhibitor, olaparib, in combination with cytotoxic chemotherapy in pediatric solid tumors. Pediatr. Blood Cancer 61, 145–150. 10.1002/pbc.24697 24038812 PMC3849815

[B135] Oehme I. Deubzer H. E. Wegener D. Pickert D. Linke J.-P. Hero B. (2008). Histone deacetylase 8 in neuroblastoma tumorigenesis. Clin. Cancer Res. 15, 91–99. 10.1158/1078-0432.CCR-08-0684 19118036

[B136] Olsson M. Beck S. Kogner P. Martinsson T. Carén H. (2016). Genome-wide methylation profiling identifies novel methylated genes in neuroblastoma tumors. Epigenetics 11, 74–84. 10.1080/15592294.2016.1138195 26786290 PMC4846113

[B137] Pai P. Reddy Y. Das I. Venkidesh B. S. Bhandari P. Rao P. (2025). Targeting neuroblastoma with hydroxamic acid based HDAC1 and HDAC2 inhibitors: insights from *in vitro* and *in vivo* studies. Invest New Drugs 43, 780–791. 10.1007/s10637-025-01559-y 40640468 PMC12515225

[B138] Pandey V. Sharma S. Pokharel Y. R. (2025). Exploring CRISPR-Cas: the transformative impact of gene editing in molecular biology. Mol. Ther. Nucleic Acids 36, 102717. 10.1016/j.omtn.2025.102717 41069986 PMC12506487

[B139] Parodi F. Carosio R. Ragusa M. Di Pietro C. Maugeri M. Barbagallo D. (2016). Epigenetic dysregulation in neuroblastoma: a tale of miRNAs and DNA methylation. Biochim. Biophys. Acta 1859, 1502–1514. 10.1016/j.bbagrm.2016.10.006 27751904

[B140] Pearson A. D. J. Federico S. Gatz S. A. Ortiz M. Lesa G. Scobie N. (2023). Paediatric strategy forum for medicinal product development of DNA damage response pathway inhibitors in children and adolescents with cancer: ACCELERATE in collaboration with the european medicines agency with participation of the food and drug administration. Eur. J. Cancer 190, 112950. 10.1016/j.ejca.2023.112950 37441939

[B141] Peifer M. Hertwig F. Roels F. Dreidax D. Gartlgruber M. Menon R. (2015). Telomerase activation by genomic rearrangements in high-risk neuroblastoma. Nature 526, 700–704. 10.1038/nature14980 26466568 PMC4881306

[B142] Plantaz D. Vandesompele J. Van Roy N. Lastowska M. Bown N. Combaret V. (2001). Comparative genomic hybridization (CGH) analysis of stage 4 neuroblastoma reveals high frequency of 11q deletion in tumors lacking MYCN amplification. Int. J. Cancer 91, 680–686. 10.1002/1097-0215(200002)9999:9999<::aid-ijc1114>3.0.co;2-r 11267980

[B143] Pugh T. J. Morozova O. Attiyeh E. F. Asgharzadeh S. Wei J. S. Auclair D. (2013). The genetic landscape of high-risk neuroblastoma. Nat. Genet. 45, 279–284. 10.1038/ng.2529 23334666 PMC3682833

[B144] Qadi S. A. Hassan M. A. Sheikh R. A. Baothman O. A. Zamzami M. A. Choudhry H. (2019). Thymoquinone-induced reactivation of tumor suppressor genes in cancer cells involves epigenetic mechanisms. Epigenet Insights 12, 2516865719839011. 10.1177/2516865719839011 31058255 PMC6452588

[B145] Qiu L. Ma Z. Wu X. (2024). Mutant p53-Mediated tumor secretome: bridging tumor cells and stromal cells. Genes 15, 1615. 10.3390/genes15121615 39766882 PMC11675497

[B146] Rabaan A. A. AlSaihati H. Bukhamsin R. Bakhrebah M. A. Nassar M. S. Alsaleh A. A. (2023). Application of CRISPR/Cas9 technology in cancer treatment: a future direction. Curr. Oncol. 30, 1954–1976. 10.3390/curroncol30020152 36826113 PMC9955208

[B147] Rader J. Russell M. R. Hart L. S. Nakazawa M. S. Belcastro L. T. Martinez D. (2013). Dual CDK4/CDK6 inhibition induces cell cycle arrest and senescence in neuroblastoma. Clin. Cancer Res. 19, 6173–6182. 10.1158/1078-0432.CCR-13-1675 24045179 PMC3844928

[B148] Rao D. Wang T. Fu C. Luo Y. Lu J. Sun Z. (2025). From intracellular drivers to immune modulators: emerging paradigms in oncogenic pathway-directed immunotherapy optimization. Cell Commun. Signal 23, 451. 10.1186/s12964-025-02465-9 41126244 PMC12542028

[B149] Rehrauer H. Wu L. Blum W. Pecze L. Henzi T. Serre-Beinier V. (2018). How asbestos drives the tissue towards tumors: YAP activation, macrophage and mesothelial precursor recruitment, RNA editing, and somatic mutations. Oncogene 37, 2645–2659. 10.1038/s41388-018-0153-z 29507420 PMC5955862

[B150] Rein D. T. Breidenbach M. Curiel D. T. (2006). Current developments in adenovirus-based cancer gene therapy. Future Oncol. 2, 137–143. 10.2217/14796694.2.1.137 16556080 PMC1781528

[B151] Rettig I. Koeneke E. Trippel F. Mueller W. C. Burhenne J. Kopp-Schneider A. (2015). Selective inhibition of HDAC8 decreases neuroblastoma growth *in vitro* and *in vivo* and enhances retinoic acid-mediated differentiation. Cell Death Dis. 6, e1657. 10.1038/cddis.2015.24 25695609 PMC4669789

[B152] Rezaei O. Honarmand Tamizkar K. Hajiesmaeili M. Taheri M. Ghafouri-Fard S. (2021). Non-coding RNAs participate in the pathogenesis of neuroblastoma. Front. Oncol. 11, 617362. 10.3389/fonc.2021.617362 33718173 PMC7945591

[B153] Rihani A. Vandesompele J. Speleman F. Van Maerken T. (2015). Inhibition of CDK4/6 as a novel therapeutic option for neuroblastoma. Cancer Cell Int. 15, 76. 10.1186/s12935-015-0224-y 26225123 PMC4518532

[B154] Rodriguez-Lopez A. M. Xenaki D. Eden T. O. Hickman J. A. Chresta C. M. (2001). MDM2 mediated nuclear exclusion of p53 attenuates etoposide-induced apoptosis in neuroblastoma cells. Mol. Pharmacol. 59, 135–143. 10.1124/mol.59.1.135 11125034

[B155] Roeschert I. Poon E. Henssen A. G. Garcia H. D. Gatti M. Giansanti C. (2021). Combined inhibition of Aurora-A and ATR kinase results in regression of MYCN-amplified neuroblastoma. Nat. Cancer 2, 312–326. 10.1038/s43018-020-00171-8 33768209 PMC7610389

[B156] Rossi A. Zacchi F. Reni A. Rota M. Palmerio S. Menis J. (2024). Progresses and pitfalls of epigenetics in solid tumors clinical trials. Int. J. Mol. Sci. 25, 11740. 10.3390/ijms252111740 39519290 PMC11546921

[B157] Rubio-San-Simón A. Marshall L. V. Doz F. Mora J. Bielamowicz K. Corradini N. (2025). Idasanutlin in combination with chemotherapy or venetoclax in pediatric and young adult patients with relapsed/refractory solid tumors (iMATRIX idasa): results of a phase I/II, multicenter, multi-arm study. Target Oncol. 21, 87–102. 10.1007/s11523-025-01186-w 41379293

[B158] Russell M. R. Penikis A. Oldridge D. A. Alvarez-Dominguez J. R. McDaniel L. Diamond M. (2015). CASC15-S is a tumor suppressor lncRNA at the 6p22 neuroblastoma susceptibility locus. Cancer Res. 75, 3155–3166. 10.1158/0008-5472.CAN-14-3613 26100672 PMC4526355

[B159] Epp S. Chuah S. M. Halasz M. (2023). Epigenetic dysregulation in MYCN-amplified neuroblastoma. Int. Journal Molecular Sciences 24. 10.3390/ijms242317085 PMC10707345 38069407

[B160] Sainero-Alcolado L. Sjöberg Bexelius T. Santopolo G. Yuan Y. Liaño-Pons J. Arsenian-Henriksson M. (2024). Defining neuroblastoma: from origin to precision medicine. Neuro Oncol. 26, 2174–2192. 10.1093/neuonc/noae152 39101440 PMC11630532

[B161] Salvati A. Melone V. Giordano A. Lamberti J. Palumbo D. Palo L. (2025). Multi-omics based and AI-driven drug repositioning for epigenetic therapy in female malignancies. J. Transl. Med. 23, 837. 10.1186/s12967-025-06856-x 40713639 PMC12297790

[B162] Schafer E. S. Rau R. E. Berg S. L. Liu X. Minard C. G. Bishop A. J. R. (2020). Phase 1/2 trial of talazoparib in combination with temozolomide in children and adolescents with refractory/recurrent solid tumors including ewing sarcoma: a children’s oncology group phase 1 consortium study (ADVL1411). Pediatr. Blood Cancer 67, e28073. 10.1002/pbc.28073 31724813 PMC9134216

[B163] Schäffer A. A. Chung Y. Kammula A. V. Ruppin E. Lee J. S. (2024). A systematic analysis of the landscape of synthetic lethality-driven precision oncology. Med 5, 73–89.e9. 10.1016/j.medj.2023.12.009 38218178

[B164] Seliger B. (2019). Combinatorial approaches with checkpoint inhibitors to enhance anti-tumor immunity. Front. Immunol. 10, 999. 10.3389/fimmu.2019.00999 31178856 PMC6538766

[B165] Seront E. Pinto A. Bouzin C. Bertrand L. Machiels J.-P. Feron O. (2013). PTEN deficiency is associated with reduced sensitivity to mTOR inhibitor in human bladder cancer through the unhampered feedback loop driving PI3K/Akt activation. Br. J. Cancer 109, 1586–1592. 10.1038/bjc.2013.505 23989949 PMC3777009

[B166] Shah P. A. Wiman K. G. Cichowski K. Rodon Ahnert J. (2025). Capturing unicorns: targeting cancers with TP53 mutations, RAS alterations beyond G12C, and MTAP loss—no target is out of the realm of possibility. Am. Soc. Clin. Oncol. Educ. Book 45, e473616. 10.1200/EDBK-25-473616 40435431

[B167] Shim J. Goldsmith K. C. (2021). A new player in neuroblastoma: YAP and its role in the neuroblastoma microenvironment. Cancers (Basel) 13, 4650. 10.3390/cancers13184650 34572875 PMC8472533

[B168] Shohet J. M. (2012). Redefining functional MYCN gene signatures in neuroblastoma. Proc. Natl. Acad. Sci. 109, 19041–19042. 10.1073/pnas.1217598109 23139408 PMC3511077

[B169] Shokouhfar M. Darzi A. Ameli F. Nami M. T. Khorasani S. K. Eini P. (2026). Immunotherapeutic advances in pediatric neuroblastoma: overcoming resistance through biomarker-guided combinations. Biomed. & Pharmacother. 196, 119020. 10.1016/j.biopha.2026.119020 41621264

[B170] Smiles W. J. Catalano L. Stefan V. E. Weber D. D. Kofler B. (2023). Metabolic protein kinase signalling in neuroblastoma. Mol. Metab. 75, 101771. 10.1016/j.molmet.2023.101771 37414143 PMC10362370

[B171] Southgate H. E. D. Chen L. Curtin N. J. Tweddle D. A. (2020). Targeting the DNA damage response for the treatment of high risk neuroblastoma. Front. Oncol. 10, 371. 10.3389/fonc.2020.00371 32309213 PMC7145987

[B172] Stambolic V. Suzuki A. de la Pompa J. L. Brothers G. M. Mirtsos C. Sasaki T. (1998). Negative regulation of PKB/Akt-dependent cell survival by the tumor suppressor PTEN. Cell 95, 29–39. 10.1016/s0092-8674(00)81780-8 9778245

[B173] Sun C. Chu A. Song R. Liu S. Chai T. Wang X. (2023). PARP inhibitors combined with radiotherapy: are we ready? Front. Pharmacol. 14, 1234973. 10.3389/fphar.2023.1234973 37954854 PMC10637512

[B174] Szydzik J. Lind D. E. Arefin B. Kurhe Y. Umapathy G. Siaw J. T. (2021). ATR inhibition enables complete tumour regression in ALK-driven NB mouse models. Nat. Commun. 12, 6813. 10.1038/s41467-021-27057-2 34819497 PMC8613282

[B175] Takagi M. Ogawa C. Iehara T. Aoki-Nogami Y. Ishibashi E. Imai M. (2022). First phase 1 clinical study of olaparib in pediatric patients with refractory solid tumors. Cancer 128, 2949–2957. 10.1002/cncr.34270 35593736

[B176] Takita J. (2021). Molecular basis and clinical features of neuroblastoma. JMA J. 4, 321–331. 10.31662/jmaj.2021-0077 34796286 PMC8580727

[B177] Thombare K. Vaid R. Pucci P. Ihrmark Lundberg K. Ayyalusamy R. Baig M. H. (2024). METTL3/MYCN cooperation drives neural crest differentiation and provides therapeutic vulnerability in neuroblastoma. EMBO J. 43, 6310–6335. 10.1038/s44318-024-00299-8 39528654 PMC11649786

[B178] Tsilingiri K. Chalari A. Christopoulou G. Voutsina A. Constantoulakis P. Potaris Κ. (2024). Genomic scarring score predicts the response to PARP inhibitors in non-small cell lung cancer. Npj Precis. Onc. 8, 291. 10.1038/s41698-024-00777-6 PMC11671589 39725687

[B179] van Groningen T. Koster J. Valentijn L. J. Zwijnenburg D. A. Akogul N. Hasselt N. E. (2017). Neuroblastoma is composed of two super-enhancer-associated differentiation states. Nat. Genet. 49, 1261–1266. 10.1038/ng.3899 28650485

[B180] van Tilburg C. M. Pfaff E. Pajtler K. W. Langenberg K. P. S. Fiesel P. Jones B. C. (2021). The pediatric precision oncology INFORM registry: clinical outcome and benefit for patients with very high-evidence targets. Cancer Discov. 11, 2764–2779. 10.1158/2159-8290.CD-21-0094 34373263 PMC9414287

[B181] Verhoeven B. M. Mei S. Olsen T. K. Gustafsson K. Valind A. Lindström A. (2022). The immune cell atlas of human neuroblastoma. CR Med. 3, 100657. 10.1016/j.xcrm.2022.100657 PMC9245004 35688160

[B182] Vernooij L. Bate-Eya L. T. Alles L. K. Lee J. Y. Koopmans B. Jonus H. C. (2021). High-throughput screening identifies idasanutlin as a resensitizing drug for Venetoclax-Resistant neuroblastoma cells. Mol. Cancer Ther. 20, 1161–1172. 10.1158/1535-7163.MCT-20-0666 33850004 PMC7611269

[B183] Wang X. Yue F. (2024). Hijacked enhancer-promoter and silencer-promoter loops in cancer. Curr. Opin. Genet. Dev. 86, 102199. 10.1016/j.gde.2024.102199 38669773

[B184] Wang H. Wang X. Xu L. Zhang J. (2022). Prognostic analysis of E2F transcription factors E2F1 and E2F3 in four independent pediatric neuroblastoma cohorts. BMC Pediatr. 22, 376. 10.1186/s12887-022-03424-w 35764946 PMC9241263

[B185] Wang L. Wang C. Sarwar Md. S. Chou P. Wang Y. Su X. (2022). PTEN-Knockout regulates metabolic rewiring and epigenetic reprogramming in prostate cancer and chemoprevention by triterpenoid ursolic acid. FASEB J. 36, e22626. 10.1096/fj.202201195R 36305462 PMC9703918

[B186] Wang C. Tan J. Y. M. Chitkara N. Bhatt S. (2024). TP53 mutation-mediated immune evasion in cancer: mechanisms and therapeutic implications. Cancers (Basel) 16, 3069. 10.3390/cancers16173069 39272927 PMC11393945

[B187] Wang X. Xu G. Ma H. Deng X. Ma G. (2025). Emerging frontiers in epigenetic-targeted therapeutics for pediatric neuroblastoma. Front. Immunol. 16, 1637626. 10.3389/fimmu.2025.1637626 40787450 PMC12331715

[B188] Wang Y. Chen W. Wang Z. Cai S. Zhao X. Jin J. (2025). Deciphering metabolic reprogramming of immune cells within the tumor microenvironment. J. Transl. Med. 23, 1055. 10.1186/s12967-025-07069-y 41053742 PMC12502548

[B189] Xie Y. Xiao D. Li D. Peng M. Peng W. Duan H. (2024). Combined strategies with PARP inhibitors for the treatment of BRCA wide type cancer. Front. Oncol. 14, 1441222. 10.3389/fonc.2024.1441222 39156700 PMC11327142

[B190] Xie X. Liu W. Yuan Z. Chen H. Mao W. (2025). Bridging epigenomics and tumor immunometabolism: molecular mechanisms and therapeutic implications. Mol. Cancer 24, 71. 10.1186/s12943-025-02269-y 40057791 PMC11889836

[B191] Xu D. Zhong J. Zeng Y. Zhang X. Wang C. Luo C. (2025). An updated patent review of TEAD modulators (2022–present). Expert Opin. Ther., 1–27. 10.1080/13543776.2025.2522747 40536362

[B192] Yamamoto N. Ishizawa K. Umemoto M. Nishimura A. Fujikawa T. Inoue S. (2025). Evaluation of minimal residual disease in patients with neuroblastoma. Mol. Diagn Ther. 29, 443–452. 10.1007/s40291-025-00788-4 40450177 PMC12227348

[B193] Yu Y. Chen F. Yang Y. Jin Y. Shi J. Han S. (2019). lncRNA SNHG16 is associated with proliferation and poor prognosis of pediatric neuroblastoma. Int. J. Oncol. 55, 93–102. 10.3892/ijo.2019.4813 31180520 PMC6561620

[B194] Yu X. Zhao H. Wang R. Chen Y. Ouyang X. Li W. (2024). Cancer epigenetics: from laboratory studies and clinical trials to precision medicine. Cell Death Discov. 10, 28. 10.1038/s41420-024-01803-z 38225241 PMC10789753

[B195] Yu W. Biyik-Sit R. Uzun Y. Chen C.-H. Thadi A. Sussman J. H. (2025). Longitudinal single-cell multiomic atlas of high-risk neuroblastoma reveals chemotherapy-induced tumor microenvironment rewiring. Nat. Genet. 57, 1142–1154. 10.1038/s41588-025-02158-6 40229600 PMC12081299

[B196] Yuan C. Li C. Lu J. Liao S. Wu R. Li D. (2025). MS275 inhibits neuroblastoma cell growth by mediating H3K27ac/PROX1 axis *in silico* and *in vitro* . FASEB J. 39, e70797. 10.1096/fj.202500464RR 40601211 PMC12219463

[B197] Yuan L. Xiong Y. Zhang Y. Gu S. Lei Y. (2026). Epigenome editing based treatment: progresses and challenges. Mol. Ther. 34, 46–67. 10.1016/j.ymthe.2025.08.047 40898613 PMC12925790

[B198] Zafar A. Wang W. Liu G. Xian W. McKeon F. Zhou J. (2021). Targeting the p53-MDM2 pathway for neuroblastoma therapy: rays of hope. Cancer Lett. 496, 16–29. 10.1016/j.canlet.2020.09.023 33007410 PMC8351219

[B199] Zahraeifard S. Xiao Z. So J. Y. Ahad A. Montoya S. Park W. Y. (2024). Loss of tumor suppressors promotes inflammatory tumor microenvironment and enhances LAG3+T cell mediated immune suppression. Nat. Commun. 15, 5873. 10.1038/s41467-024-50262-8 38997291 PMC11245525

[B200] Zanconato F. Cordenonsi M. Piccolo S. (2016). YAP/TAZ at the roots of cancer. Cancer Cell 29, 783–803. 10.1016/j.ccell.2016.05.005 27300434 PMC6186419

[B201] Zeineldin M. Patel A. G. Dyer M. A. (2022). Neuroblastoma: when differentiation goes awry. Neuron 110, 2916–2928. 10.1016/j.neuron.2022.07.012 35985323 PMC9509448

[B202] Zhang J. Zhang Y. Lin X. Han X. Meredith K. L. Li Z. (2023). The effects of the tumor suppressor gene PTEN on the proliferation and apoptosis of breast cancer cells *via* AKT phosphorylation. Transl. Cancer Res. 12, 1863–1872. 10.21037/tcr-23-826 37588750 PMC10425639

[B203] Zhang W. Zhang M. Sun M. Hu M. Yu M. Sun J. (2023). Metabolomics-transcriptomics joint analysis: unveiling the dysregulated cell death network and developing a diagnostic model for high-grade neuroblastoma. Front. Immunol. 14, 1345734. 10.3389/fimmu.2023.1345734 38239355 PMC10794662

[B204] Zhao S. J. Prior D. Heske C. M. Vasquez J. C. (2024). Therapeutic targeting of DNA repair pathways in pediatric extracranial solid tumors: current state and implications for immunotherapy. Cancers 16, 1648. 10.3390/cancers16091648 38730598 PMC11083679

[B205] Zhu K. Su F. Yang J. Xiao R. Wu R. Cao M. (2024). TP53 to mediate immune escape in tumor microenvironment: an overview of the research progress. Mol. Biol. Rep. 51, 205. 10.1007/s11033-023-09097-7 38270700 PMC10811008

